# *Staphylococcus epidermidis* WF2R11 Suppresses PM_2.5_-Mediated Activation of the Aryl Hydrocarbon Receptor in HaCaT Keratinocytes

**DOI:** 10.1007/s12602-022-09922-8

**Published:** 2022-06-21

**Authors:** Eulgi Lee, Hyeok Ahn, Shinyoung Park, Gihyeon Kim, Hyun Kim, Myung-Giun Noh, Yunjae Kim, Jae-sung Yeon, Hansoo Park

**Affiliations:** 1 Department of Biomedical Science and Engineering, Gwangju Institute of Science and Technology (GIST), Gwangju, 61005 Republic of Korea; 2 Genome and Company, Pangyo-ro 253, Bundang-gu, Seoungnam-si, Gyeonggi-do 13486 Republic of Korea

**Keywords:** Particulate matter, Keratinocyte, Aryl hydrocarbon receptor, *Staphylococcus epidermidis*, Skin microbiome

## Abstract

The skin supports a diverse microbiome whose imbalance is related to skin inflammation and diseases. Exposure to fine particulate matter (PM_2.5_), a major air pollutant, can adversely affect the skin microbiota equilibrium. In this study, the effect and mechanism of PM_2.5_ exposure in HaCaT keratinocytes were investigated. PM_2.5_ stimulated the aryl hydrocarbon receptor (AhR) to produce reactive oxygen species (ROS) in HaCaT cells, leading to mitochondrial dysfunction and intrinsic mitochondrial apoptosis. We observed that the culture medium derived from a particular skin microbe, *Staphylococcus epidermidis* WF2R11, remarkably reduced oxidative stress in HaCaT cells caused by PM_2.5_-mediated activation of the AhR pathway. *Staphylococcus epidermidis* WF2R11 also exhibited inhibition of ROS-induced inflammatory cytokine secretion. Herein, we demonstrated that *S. epidermidis* WF2R11 could act as a suppressor of AhRs, affect cell proliferation, and inhibit apoptosis. Our results highlight the importance of the clinical application of skin microbiome interventions in the treatment of inflammatory skin diseases.

**Supplementary Information:**

The online version contains supplementary material available at 10.1007/s12602-022-09922-8.

## Introduction

Air pollution from particulate matter (PM) has detrimental effects on humans and animals [1]. Industries, cars, coal-fired power plants, and other anthropogenic activities contribute to air pollution, adversely affecting the environment and human health [2]. The US Environmental Protection Agency (EPA) classified PM according to particle size as PM_0.1_ (ultrafine, ≤ 0.1 µm), PM_2.5_ (fine, ≤ 2.5 µm), and PM_10_ (coarse, ≤ 10 µm) [3]. PM_0.1_ and PM_2.5_ are the most prevalent among all types of PM. The adhesion of contaminants, oxidizing gases, organic compounds, or transition metals to PM_0.1_ and PM_2.5_ introduces toxins to the body [4, 5].

The skin, the largest organ of the human body, is a multi-layered structure comprising the epidermis, dermis, and subcutaneous tissues [6, 7]. It is the primary barrier against external contaminants and serves as a repository for millions of microorganisms [8]. The composition of the skin microbial community depends upon the site and the presence of external irritants [9]. For instance, differences in fatty acid concentrations in the composition of a specific skin site cause changes in the abundance of bacterial species [10–13]. In a lipophilic environment, *Propionibacterium* species dominate, whereas, in moist areas, *Staphylococcus* and *Corynebacterium* species dominate. An imbalance in the composition of bacterial species—i.e., dysbiosis—destroys the diversity of the microbial community, causing deterioration and inflammation of the skin and a plethora of skin disorders [12, 14–16]. Airborne PM_2.5_ can cause severe skin diseases, including eczema, upon permeating the human skin cells [17]. However, alterations in skin microbiomes as a result of PM_2.5_-induced dermatitis have not been evaluated.

PAHs contained in PM_2.5_ mediate many biological effects, including carcinogenicity and developmental defects [18–20]. Several PAHs are direct activators of the aryl hydrocarbon receptor (AhR) and may subsequently play a crucial role in the induction and action of cytochrome P450 (CYP) 1A1 and 1B1 [19, 21]. Most PAHs are not water-soluble, but substances such as anthracene, benz[a]anthracene, and benzo[a]pyrene are capable of secondary oxidation to water-soluble oxygen metabolites (oxy-PAH,) [22–24]. These PAHs can act as potent regulators of CYP1 family enzymes and trigger the activation of AhR and sub-mechanisms (Table 1) [25]. Therefore, *CYP1* and *Cox-2* are deemed important functional biomarkers involved in AhR-mediated signaling. In addition to the PAHs constituting PM_2.5_, PM_2.5_ comprises various transition metals and organic compounds, which can induce excessive ROS production in mitochondria and cell cytoplasm [26]. It induces oxidative stress directly in the epidermis and dermis, triggers inflammation, and affects cell proliferation and apoptosis [27].

**Table 1:** Certified mass fraction values for PAHs in urban particulate SRM 1648a

**PAHs**	**Mass fraction** **(mg/kg)**	**Water-soluble** **(at 25 °C)**	**AhR** **regulation**	**CYP regulation**
**Phenanthrene**	4.86 ± 0.17	Soluble (1.15 mg/L)	Activation	Activation
2-Methylphenathrene	0.96 ± 0.12	Insoluble		
3-Methylphenathrene	0.614 ± 0.067	Insoluble		
**Fluoranthene**	8.07 ± 0.14	Soluble (265 μg/L)	Activation	Inhibition
Pyrene	5.88 ± 0.07	Insoluble		
Benzo[g,h,i]fluoranthene	1.17 ± 0.05	Insoluble		
Benz[a]anthracene	2.71 ± 0.15	Insoluble		
Chrysene	6.12 ± 0.06	Insoluble		
Triphenylene	2.04 ± 0.13	Soluble (6.6 μg/L)		
Benzo[k]fluoranthene	3.03 ± 0.24	Insoluble		
Benzo[e]pyrene	4.85 ± 0.07	Soluble (0.2 to 6.2 μg/L)		
**Benzo[a]pyrene**	2.57 ± 0.10	Soluble (0.2 to 6.2 μg/L)	Activation	Activation
Perylene	0.742 ± 0.048	Nearly insoluble		
Benzo[g,h,i]perylene	5.00 ± 0.18	Insoluble		
Indeno[1,2,3,-cd]pyrene	4.17 ± 0.17	Insoluble		
**Dibenz[a,j]anthracene**	0.407 ± 0.039	Soluble (0.00166 mg/L)	Activation	Activation
Benzo[b]chrysene	0.405 ± 0.041	Insoluble		
Picene	0.586 ± 0.058	Insoluble		
**Coronene**	2.28 ± 0.10	Soluble (0.14 μg/L)	Activation	Activation
Dibenzo[b,k]fluoranthene	0.947 ± 0.054	Insoluble		
Dibenzo[a,e]pyrene	0.622 ± 0.045	Insoluble		

Simultaneously, increased free radical production in skin tissue stimulates the release of inflammatory cytokines, such as tumor necrosis factor-alpha (TNF-α) and interleukins (ILs). These cytokines induce activated neutrophil infiltration and phagocytic production of radicals [28, 29]. Excessive accumulation of ROS triggers apoptosis through Cox-2 and TNF-α. Nuclear factor-kappa B (NF-κB) translocation to the nucleus induces apoptosis by downregulating Bcl-2, upregulating Bax, releasing cytochrome-c from mitochondria, and activating caspases [30–32]. We investigated whether *Staphylococcus epidermidis* WF2R11, a member of the human skin microbiome, alleviates the oxidative stress generated due to PM_2.5_-mediated ROS accumulation, and whether *S. epidermidis* WF2R11 inhibits PM_2.5_-induced apoptosis in human skin keratinocytes (HaCaT) in vitro.

## Methods

### AhR Complex Gene Set Score Analysis of Gene Expression Data from Public Resources

All processed gene expression data used in this study were procured from the Gene Expression Omnibus repository with accession number GSE107871 [30]. This RNA-seq dataset contained 24 samples, which consisted of the lesion site of a psoriasis patient, non-lesion area of the psoriasis patient, and skin from normal control groups [30]. The processes of patient recruitment, sampling, and RNA sequencing have been previously described by Mohan [30]. To calculate the AhR score, gene set variation analysis (GSVA) was performed using the AhR complex gene set of harmonizome databases using the GSVA package in R Project 4.2.0 program [33]. In the GSVA package, the “ssgsea” method was used, and the analysis was carried out between a minimum size of 10 and a maximum size of 500 [34].

### Human Skin Sample Collection and Preparation

The study for the isolation of permanent bacteria on the skin was approved by the Institutional Review Board (IRB; P01-201,605–31-003) of Korea National Institute for Bioethics Policy (KONIBP). All study protocols adhered to relevant ethical guidelines. In addition, all participants provided written informed consent before enrolment. In addition, this study protocols adhered to relevant ethical guidelines and regulations. Skin samples were collected from donors who had no history of skin disease. Before collecting the skin samples, the participants were asked not to wash their faces for longer than 12 h. Samples were obtained by rubbing the donor’s face for 1 min or 20 times vigorously with a sterilized cotton swab soaked in distilled water and were then placed in a 10-mL trophic soy broth (TSB) solution.

### Microbial Sample Isolation and 16S rRNA PCR Amplification and Sequencing

The skin samples collected from ten donors were diluted to 10^–1^–10^–threefold^ using phosphate-buffered saline (PBS). Then, 100 μL of each diluted solution was spread onto R2A, TSB, Luria–Bertani, MRS (De Man, Rogosa, and Sharpe), and blood (Columbia agar with 5% sheep blood) agar plates (Bio-Rad, Hercules, CA, USA). The inoculated plates were incubated at 37 °C for up to 72 h; whereafter, the single colonies on the plates were picked up, and their 16S rRNA genes were amplified using colony PCR. The applied parameters for the PCR were as follows: initial denaturation at 95 °C for 15 min, then 32 cycles of denaturation at 95 °C for 30 s, annealing at 55 °C for 30 s, and extension at 72 °C for 1 min and 45 s, and then a final extension step at 72 °C for 5 min. The primers for PCR were 27F (5ʹ-AGAGTTTGATCCTGGCTCAG-3ʹ) and 1492R (5ʹ-GGTTACCTTGTTACGACTT-3ʹ). Purification of the amplified DNA was carried out using the EZ-pure PCR Purification Kit (Ver 2, Enzynomics, Daejeon, South Korea), and the nucleotide sequences of the genes were determined using the ABI 3730xl system (Macrogen, Seoul, South Korea). For phylogenetic analysis, the 16S rRNA gene sequences were analyzed using the nucleotide BLAST program available at the NCBI website (https://blast.ncbi.nlm.nih.gov/Blast.cgi?PROGRAM=blastn&PAGE_TYPE=BlastSearch&LINK_LOC=blasthome).

### Hemolysis Tests for Isolated Bacteria

For hemolysis testing of the isolated bacteria, 73 single colonies were obtained by streaking bacterial samples onto TSB plates using an inoculation loop. The colonies were re-streaked onto blood agar plates (trypticase soy agar with 5% sheep blood) and incubated at 37 °C. The color of the colonies, indicating the hemolysis pattern, was noted at 48 h. From the isolation and subsequent analysis of 16S rRNA gene sequences, 50 species and 135 strains, including a variety of skin bacteria, were identified (data not shown). Hemolysis analysis of the isolated bacteria showed that most of the microbes had a β (35 strains) or γ pattern (37 strains), and the α pattern (1 strain) was not frequently observed (data not shown). For further analyses, six strains that showed γ (gamma) hemolysis patterns were selected.

### Preparation of PM_2.5_

Urban PM NIST 1648a (PM_2.5_) was purchased from Sigma-Aldrich, St. Louis, MO, USA. The PM_2.5_ dose used in this study was established based on US EPA air quality standards for particulate pollution and AQI (Air Quality Index) revisions. The composition and AQI category of Urban PM NIST 1648a are indicated in Table 1. PM_2.5_ stock solutions (50, 100, and 200 μg/mL) were prepared in DMEM and sonicated for 10 min to avoid agglomeration of the suspended PM_2.5_ particles. All experiments were performed within 1 h of stock preparation to avoid variability in PM_2.5_ compositions in the solution.

### Cell Culture and Treatment of PM_2.5_ with Se Solution

HaCaT cells were purchased from PromoCell (Heidelberg, Germany). A total of 3 × 10^5^ HaCaT cells were maintained at 37 °C in an incubator with a humidified atmosphere of 5% CO_2_ and are tested four times per year for mycoplasma using PCR. All studies were conducted within 6 months of the latest test date (11–2020). HaCaT cells were cultured in DMEM with 10% heat-inactivated FBS and an antibiotic–antimycotic (100 units/mL penicillin, 100 µg/mL streptomycin, and 0.25 µg/mL amphotericin B) (Gibco, Thermo Fisher Scientific, Waltham, MA, USA) for no longer than 4 weeks before use. For PM_2.5_ treatment, HaCaT cells were inoculated in 6-well plates, incubated in an atmosphere of 5% CO_2_ at 37 °C, and grown till they achieved 80% confluence. After 12 h, the cells were washed once with PBS, and 2 mL of each concentration of PM_2.5_ stock solution was added to the cells, depending on the purpose of the in vitro assays. Then, 10% of conditioned *S. epidermidis* WF2R11 medium (i.e., Se solution) was added to the cells together with a supplement-free medium simultaneously as PM_2.5_ treatment.

### AhR, CYP1A1 Gene Knockdown

siRNA against human *AhR* and *CYP1A1* mRNA was commercially synthesized by Bioneer, Daejeon, South Korea: siAHR (forward, 5′-CACUCAGACUACCACACAU-3′, reverse, 5′-AUGUGUGGUAGUCUGAGUG-3′); siCYP1A1 (forward, 5′-GCUAGGGUUAGGAGGUCCU-3′, reverse, 5′-AGGACCUCCUAACCCUAGC-3′). To transfect the siRNA oligo, we used Lipofectamine™ RNAiMAX (Invitrogen, Carlsbad, CA, USA) according to the manufacturer’s instructions. Briefly, 24 h before transfection, 3 × 10^5^ HaCaT cells were seeded onto 6-well plates with 2.5 mL of growth medium without antibiotics to reach 50–60% confluence at the time of transfection. AhR and CYP1A1 siRNA were incubated with HaCaT cells for 24 h. The efficiency of gene silencing by siRNA was evaluated using real-time PCR (qPCR).

### Cell Viability Assay

HaCaT cells (3 × 10^5^) were seeded into 48-well plates and incubated for 24 h in 2 mL of the complete medium. Then, 1, 5, 10, 20, or 30% of *S. epidermidis* WF2R11 culture supernatant was added to the cells, respectively, and incubated for another 48 h. After washing the cells once with PBS, 3-(4,5-dimethylthiazol-2-yl)-2,5-diphenyltetrazolium bromide (MTT) solution was added to each well, and the plates were incubated for 4 h. Then, the medium was discarded, and dimethyl sulfoxide was added to dissolve the formazan crystals. Optical density was measured at 570 nm using a microplate reader and was normalized relative to the untreated control.

### RNA Isolation and qPCR

Total RNA was isolated from cells using TRIzol reagent (TaKaRa, Shiga, Japan) according to the manufacturer’s instructions. cDNA was synthesized from 1 μg total RNA using the Reverse Transcription Premix (Elpis-Biotech, Daejeon, South Korea) under the following reaction conditions: 45 °C for 45 min and 95 °C for 5 min. Gene expression was quantified using qPCR, and data were analyzed using the StepOne Plus™ software (Applied Biosystems, Foster City, CA, USA). qPCR amplification reactions were performed using SYBR Green PCR Master Mix with premixed ROX (Applied Biosystems, Foster City, CA, USA). The following primer pairs (Bioneer, Daejeon, South Korea) were used in the ABI 7300 Cycler internal reaction according to the manufacturer’s protocol (Online Resource 7). The reaction conditions were as follows: 40 cycles for 2 min at 50 °C, 10 min at 95 °C, 10 s at 95 °C, and 1 min at 60 °C. 18S rRNA was used as an internal control.

### Measurement of Proinflammatory Cytokine Concentrations

The proinflammatory cytokines (IL-1β, IL-6, IL-8, and IFN-γ) produced were measured using an ELISA assay. HaCaT cells (6 × 10^5^), seeded in 6-well culture plates, were pretreated with PM_2.5_ (50, 100, and 200 μg/mL) for 12 h and then treated with the supernatant of the *S. epidermidis* WF2R11 for 12 h. After the treatment period, aliquots of samples (100 μL/well) were collected from the experimental medium, and the production of cytokines (IL-1β, IL-6, IL-8, and IFN-γ) was measured using a Human ILs Quantikine ELISA Kit (R&D Systems, Minneapolis, MN, USA) according to the manufacturer’s instructions.

#### Western Blotting for Measuring Protein Expression

Total HaCaT cells protein was extracted using lysis buffer (Thermo Fisher Scientific, Walthan, MA, USA) supplemented with protease/phosphatase inhibitor cocktail on ice for 30 min. BCA kit was used to measure the concentration of the proteins and for protein quantification. Proteins (20 µg) were mixed with loading buffer, separated using 10% sodium dodecyl sulfate–polyacrylamide gel electrophoresis (SDS-PAGE), and transferred to polyvinylidene fluoride (PVDF) membranes (Bio-Rad, Hercules, CA, USA). The PVDF membranes were blocked with 5% skim milk in Tris-buffered saline with Tween (TBST) for 2 h at 37 °C and incubated with primary antibodies against β-actin (1:2000), Bax (1:1000 dilution), Bcl-2 (1:500 dilution), p38 (1:1000 dilution), p-p38 (1:1000 dilution), JNK (1:1000 dilution), p-JNK (1:1000 dilution), ERK (1:2000 dilution), and p-ERK (1:2000 dilution) at 4 °C overnight. After washing 3 times with TBST, PVDF membranes were incubated with appropriate secondary antibodies for 1 h at room temperature. The immunoblots were visualized with a chemiluminescence detection system. The optical density (OD) of western blot bands was measured using ImageJ software (National Institute of Health, Bethesda, MD, USA). The data was normalized to the level of reference proteins and then averaged and presented as a relative fold change of control, from at least three independent experiments.

### Annexin V-PI Assay for Mitochondrial Apoptosis

For Annexin V-Pi assays, HaCaT cells were stained with Annexin V-FITC and PI and assessed for mitochondrial apoptosis using flow cytometry according to the manufacturer’s protocol (Sigma-Aldrich, St. Louis, MO, USA). Early apoptotic cells (annexin V positive, PI negative), necrotic cells (annexin V positive, PI positive), and viable cells (annexin V negative, PI negative) were classified using flow cytometry and fluorescence detection of annexin V bound to HaCaT cells. Apoptotic cells were filtered and determined using flow cytometry (BD FACS Calibur, Franklin Lakes, NJ, USA). The data processing was performed using the FlowJo 7.6.1 software.

### Mitochondrial Superoxide Detection

For the measurement of mitochondrial peroxide, HaCaT cells treated with different concentrations of PM_2.5_ were detected with fluorescence microscopy using MitoSOX™ Red Mitochondrial Superoxide indicator (Thermo Fisher, St. Louis, MO, USA) as a specific fluorescence probe. HaCaT cells treated with PM_2.5_ were incubated with 5 μM of the probe for 30 min at 37 °C in the dark. Then, cells were thoroughly washed with warm Hank’s balanced salt solution (HBSS) buffer and mounted for imaging. MitoSOX™ Red-stained cells were visualized at an excitation wavelength of 510 nm and an emission wavelength of 580 nm.

### Mitochondrial Membrane Potential (Δψ_m_) Measurement

The mitochondrial membrane potential and early stage of apoptosis were analyzed via fluorescence microscopy after staining with 5,5,6,6-tetrachloro-1,1,3,3-tetraethylbenzimidazolylcarbocyanine iodide (JC-1, Invitrogen, Carlsbad, CA, USA), a lipophilic cationic fluorescence dye. JC-1 dye is characterized by green fluorescence emission at ~ 529 nm in the monomeric form of the probe, which changes to red (~ 590 nm) with the concentration-dependent formation of red J-aggregates.

### Diff-Quik Staining for Observing Cytological Features

Diff-quik (catalog no. 111661; Merck, Darmstadt, Germany) staining preparations of HaCaT cells were obtained by spreading 3 × 10^5^ normal cells onto LAB-TEK® chamber slides (Thermo Fisher, St. Louis, MO, USA), treating the cells with 100 μg/mL PM_2.5_, and incubating the slides for 24 h at 37 °C. A thin smear of each HaCaT was stained using the diff-quik staining by manufacturer’s instructions. Briefly, the air-dried smears were sequentially dipped five times in a methanol fixative solution I, five more times in stain solution II (color reagent red), then dipped five times in stain solution III (color reagent blue), and gently rinsed with distilled water. The overall staining process took ~ 40 s. The slides were then cleared in xylol and mounted in a non-aqueous mounting medium. A diff-quik-stained smear of each HaCaT slide was analyzed by a board-certified pathologist (MGN).

### Ki-67 Immunohistochemistry

HaCaT cells were prepared by spreading 3 × 10^5^ normal cells onto LAB-TEK® chamber slides (Thermo Fisher, St. Louis, MO, USA), followed by treatment with 100 μg/mL PM_2.5_, and incubation for 24 h at 37 °C. After removing the chamber wall, all the slides were fixed in neutral-buffered formalin. Endogenous peroxidase activity was blocked by incubating with 3% hydrogen peroxidase in methanol for 5 min. Epitope retrieval of Ki-67 was carried out by boiling in a pressure cooker in Tris–EDTA solution buffer at pH 9.0 for 15 min at 99 °C. After washing with PBS, the sections were incubated with the anti-human Ki-67 polyclonal antibody (ab15580, rabbit anti-human; Abcam, Cambridge, MA, USA) at a dilution of 1:600 using an antibody diluent (GBI Labs, Bothell, WA, USA) for 60 min at 23–25.5 °C. The primary antibody against Ki-67 was detected with Polink-2 Plus HRP Rabbit with DAB Kit (GBI Labs, Bothell, WA, USA) for 30 min at approximately 23–25.5 °C. Diaminobenzidine (Sigma-Aldrich, St. Louis, MO, USA) was used as the chromogen and incubated for 3 min at 25 °C. The slides were counterstained with hematoxylin. For the positive control, a normal tonsil tissue was used; for the negative control, the primary antibody was omitted, and PBS was used in each experiment. The Ki-67 index was quantified by estimating the number of positive HaCaT cells expressing nuclear Ki-67 (brown colored) among the total number of HaCaT cells. Both weakly and strongly labeled nuclei were included in the estimates of proliferating cells. All immunostained slides were evaluated twice by a blinded board-certified pathologist (MGN). A semi-quantitative assessment of the average percentage of Ki-67 positive cells in several fields comprising more than 1000 cells was performed.

### Statistical Analysis

All data were tested for normality, and the dataset was analyzed using one-way ANOVA. The post hoc analysis was then carried out using the Bonferroni test for comparison between pairs. All results are presented as the mean ± SEM. Correlations were determined using Pearson’s correlation analysis. All statistical analyses were performed using GraphPad Prism 5.02 (GraphPad Software, San Diego, CA, USA) and R-4.2.0 for Windows. The statistical significance was set at *p* < 0.05.

## Results

### PM_2.5_ Stimulates AhR to Induce ROS Production in HaCaT Cells

**Fig. 1:**
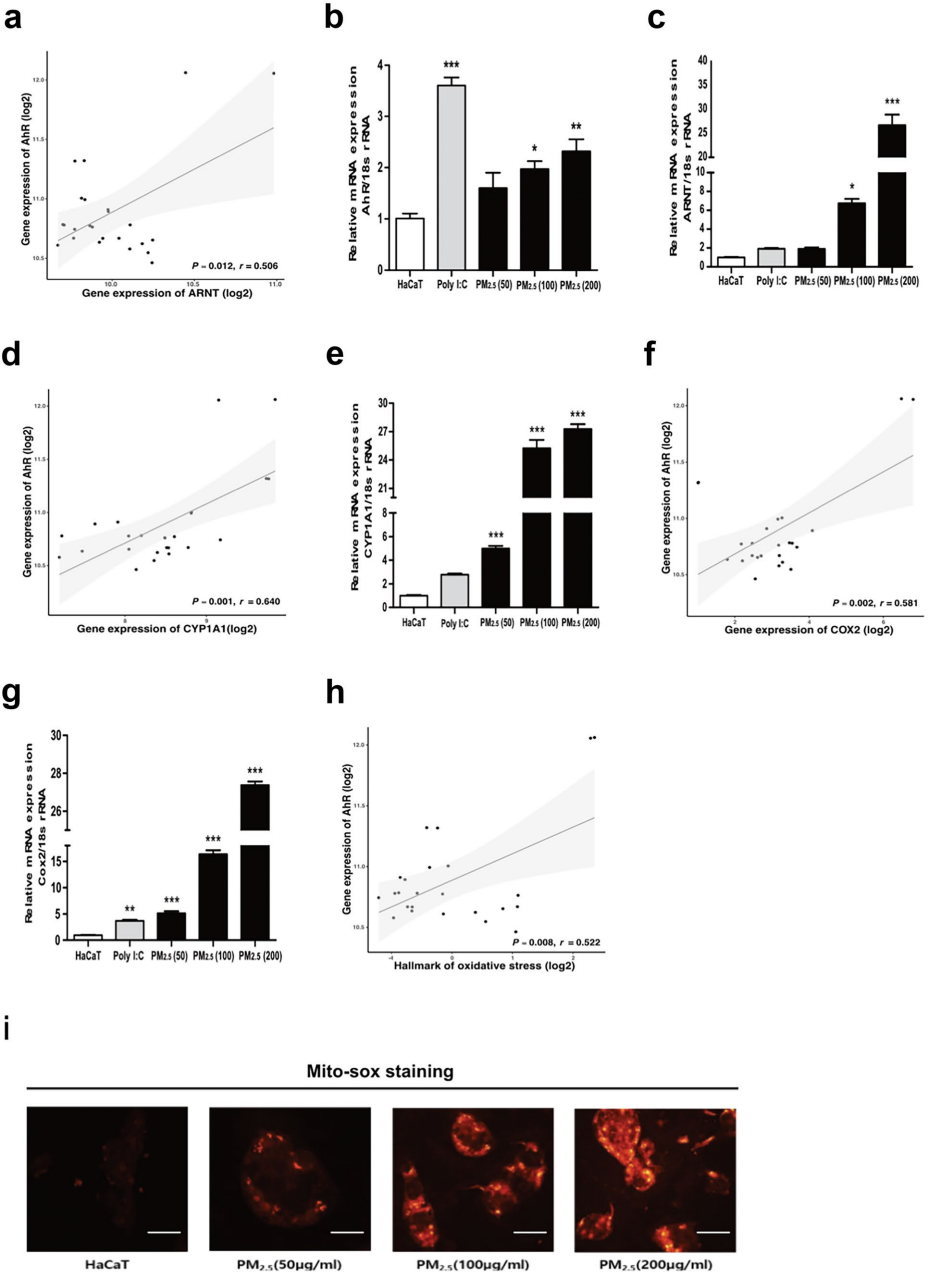
PM_2.5_ activates the AhR signaling pathway, leading to ROS production.(**a**) Correlation analysis of AhR with ARNT expression reveals their positive correlation at the mRNA level. (**b**) Measurement of the mRNA level of *AhR* relative to 18S rRNA. (**c**) Measurement of the mRNA level of *ARNT* relative to 18S rRNA. (**d**) Correlation analysis of *AhR* with *CYP1A1* expression reveals their positive correlation at the mRNA level. (**e**) Measurement of the mRNA level of *CYP1A1* relative to 18S rRNA. (**f**) Correlation analysis of *AhR* with *Cox-2* expression reveals their positive correlation at the mRNA level. (**g**) Measurement of the mRNA level of *Cox-2* relative to 18S rRNA. (**h**) Correlation analysis of AhR with the hallmark of oxidative stress reveals their positive correlation at the mRNA level. (**i**) Measurement of Mito-SOX fluorescence expression when treated with 50, 100, and 200 μg/mL of PM_2.5_ compared to that of the control HaCaT cells. Scale bar, 20 μm. The mRNA level of *AhR*, *ARNT*, *CYP1A1*, and *Cox-2* relative to 18S rRNA was measured after 12 h of PM2.5 treatment for each concentration. Correlation was determined using Pearson’s correlation analysis. Bonferroni test for comparison between pairs was used to calculate statistical significance. *p < 0.05, **p < 0.01, ***p < 0.001, or ns, non-significant; compared to normal HaCaT cells. AhR, aryl hydrocarbon receptor; ARNT, AhR nuclear translocator

AhR signaling is associated with exposure to PM_2.5_ and ROS production [35]. To reaffirm the relationship between PM_2.5_, AhR stimulation, and ROS production, we performed RNA-seq analysis of whole skin using open-access data from a study by Swindell [36]. The transcriptomic analysis involved in the AhR signaling pathway were identified to predict the association between the AhR-related genes and ROS production. We first analyzed the correlation between AhR and AhR nuclear translocator (ARNT) upon exposure to PM_2.5_. *AhR* expression was positively correlated with *ARNT* expression (*p* = 0.012, *r* = 0.506) (Fig. 1a). To prove the positive correlation of AhR-ARNT gene expression evidenced by transcriptomic analysis, after PM_2.5_ treatment in HaCaT cells, mRNA expression levels of both genes were measured. The dosage of PM_2.5_ classified each concentration of PM_2.5_ in accordance with EPA standards (Online Resource 1). Each concentration of PM_2.5_ used in the following experiments was set to moderate (PM_2.5_; 50 μg/mL), unhealthy (PM_2.5_; 100 μg/mL), hazardous (PM_2.5_; 200 μg/mL). Similar to the correlation between the two genes presented in Fig. 1a, AhR and ARNT mRNA expression significantly increased as the PM_2.5_ concentration increased, suggesting the possibility that PM_2.5_ may act as a ligand for AhR and induce the AhR/ARNT complex formation (Fig. 1b, c). As a sub-mechanism of the AhR/ARNT complex, *CYP1A1* and *Cox-2* play important roles in ROS production in the cytoplasm [37, 38]. The expression of *CYP1A1* positively correlated with *AhR* expression (*p* = 0.001, *r* = 0.640; Fig. 1d). Furthermore, the expression of *Cox-2* positively correlated with that of *AhR* (*p* = 0.002, *r* = 0.581; Fig. 1f). The mRNA expression of each of these two genes increased from approximately fivefold to over 27-fold upon exposure to PM_2.5_ at minimal and maximal concentrations of 50 μg/mL and 200 μg/mL, respectively (Fig. 1e, g). PM_2.5_ reportedly upregulates ROS production and induces oxidative stress via AhR signaling (*p* = 0.008, *r* = 0.522; Fig. 1h) [37, 39]. To observe mitochondrial ROS accumulation, HaCaT cells were treated with varying concentrations of PM_2.5_, and the ROS levels were observed using a MitoSOX fluorescent dye that specifically targets mitochondria (Fig. 1i). The increase in red fluorescence is related to the oxidation of the MitoSOX Red reagent by peroxide in the mitochondria. Therefore, as the concentration of PM_2.5_ increased, ROS accumulation in the mitochondria was observed to increase substantially.

### PM_2.5_ Induces Mitochondrial Dysfunction and Promotes Intrinsic Mitochondrial Apoptosis

**Fig. 2:**
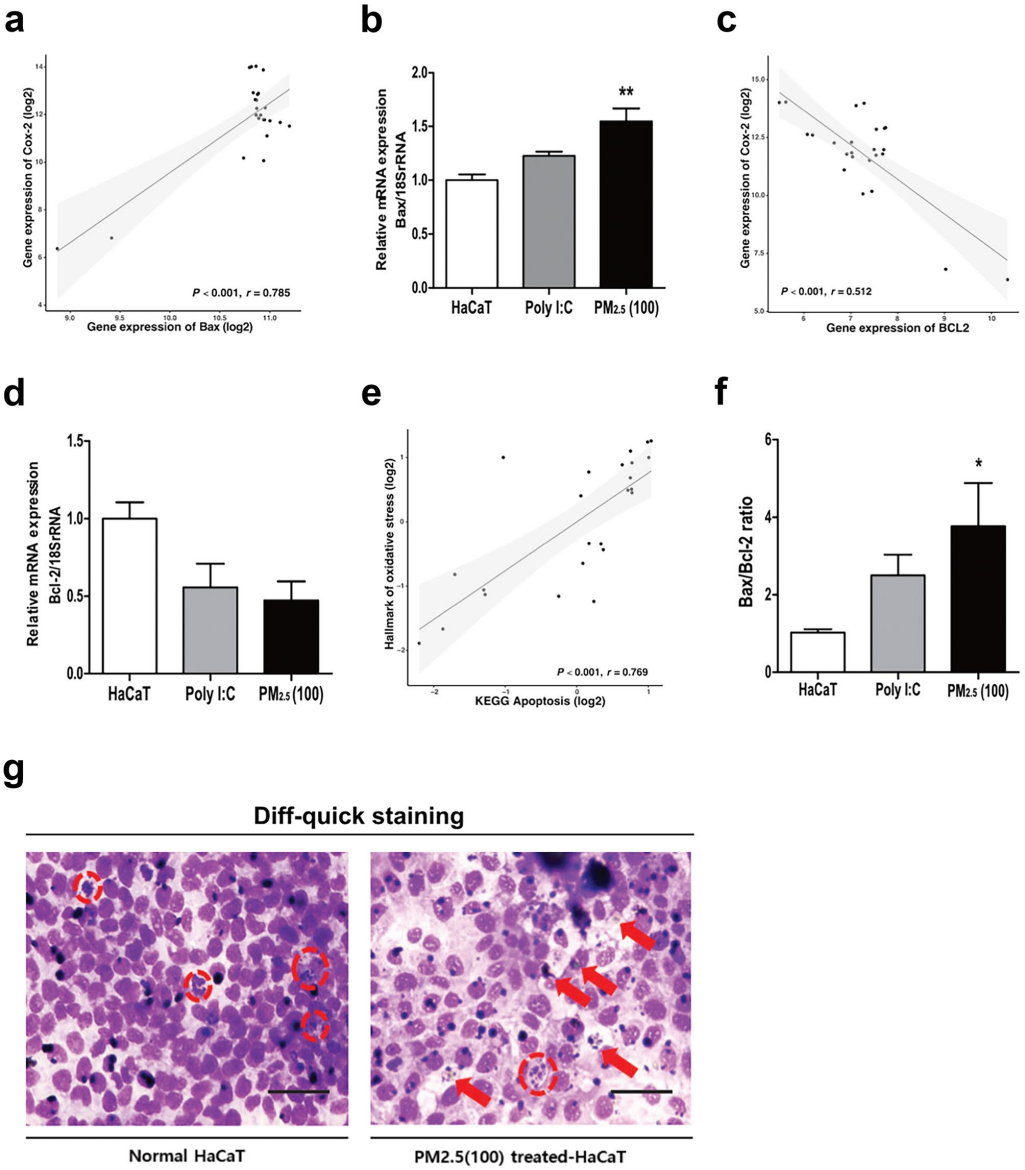
Oxidative stress caused by ROS accumulation in the cytoplasm is closely related to apoptosis.(**a**) Correlation analysis of *Cox-2* with *Bax* expression reveals their positive correlation at the mRNA level. (**b**) Measurement of the mRNA level of *Bax* relative to 18S rRNA. (**c**) Correlation analysis of *Cox-2* with *Bcl-2* expression reveals their negative correlation at the mRNA level. (**d**) Measurement of the mRNA level of *Bcl-2* relative to 18S rRNA. (**e**) Correlation analysis of hallmark genes of oxidative stress with KEGG-apoptosis reveals their positive correlation. (**f**) Measurement of the relative *Bax/Bcl-2* ratio for mitochondrial-dependent cell death after PM_2.5_ treatment. (**g**) Diff-quick staining for observing cytological features of 100 μg/mL PM_2.5_-treated group compared to control HaCaT cells (normal HaCaT: red circle, mitosis progression; PM_2.5_-treated-HaCaT: red arrows, nuclear degeneration; red circle, apoptosis). Scale bar, 50 μm. The mRNA level of *Bax* and *Bcl-2* relative to 18S rRNA was measured after 12 h of PM2.5 treatment for each concentration. Correlation was determined using Pearson’s correlation analysis. Bonferroni test for comparison between pairs was used to calculate statistical significance. *p < 0.05, **p < 0.01, ***p < 0.001, ns, non-significant; compared to normal HaCaT cells

Free radicals that have accumulated in the cytoplasm initiate oxidative damage mechanisms in various organelles, including mitochondria [31, 40]. Accumulation of cytoplasmic ROS in HaCaT cells causes mitochondrial dysfunction, increasing the expression of the mitochondrial apoptosis factor, *Bax*, and decreasing the expression of apoptosis inhibitor, *Bcl-2* [41, 42]. Thus, the increase in the ratio of *Bax/Bcl-2* via ROS is defined as a direct marker of mitochondrial damage. Using correlation analysis, *Bax* was positively correlated with the expression of *Cox-2* (*p* < 0.001, *r* = 0.785; Fig. 2a). After treatment with 100 µg/mL PM_2.5_, the mRNA level of *Bax* was significantly increased compared to that of the control HaCaT cells and the positive control polyinosinic:polycytidylic acid (poly I:C group) (Fig. 2b). Meanwhile, the expression of *Bcl-2* showed significant negative correlation with *Cox-2* expression (*p* < 0.001, *r* = 0.512) in the transcriptomic analysis (Fig. 2c). The mRNA levels of *Bcl-2* also showed an inverse trend compared to that of *Bax*. Although not significant, the reduction in *Bcl-2* mRNA levels was remarkable compared to that of the control HaCaT cells (Fig. 2d). Also, the hallmark genes of oxidative stress induced by PM_2.5_ showed a significant positive correlation with the Kyoto Encyclopedia of Genes and Genomes (KEGG) category, apoptosis (*p* < 0.001, *r* = 0.769; Fig. 2e). Compared to the normal HaCaT cells and poly I:C-treated group, the PM_2.5_-treated group was evaluated as a factor that significantly increased mitochondrial dysfunction (Fig. 2f). Furthermore, the intercellular density of the control HaCaT cells and the PM_2.5_-treated cells (100 µg/mL) was compared and analyzed. Control HaCaT cells showed high intercellular density and evidence of persistent mitosis, whereas, in the PM_2.5_-treated cells, the intercellular density was low, and indicators of nuclear degeneration as well as apoptosis were evident (Fig. 2g). As a result, an increased *Bax/Bcl-2* ratio in HaCaT cells may indicate the onset of intrinsic mitochondrial apoptosis upon exposure to PM_2.5_ [43].

### *Staphylococcus epidermidis* WF2R11 Supernatant (Se Solution) Reduces the mRNA Levels of Inflammatory Cytokines Induced via PM_2.5_

**Fig. 3:**
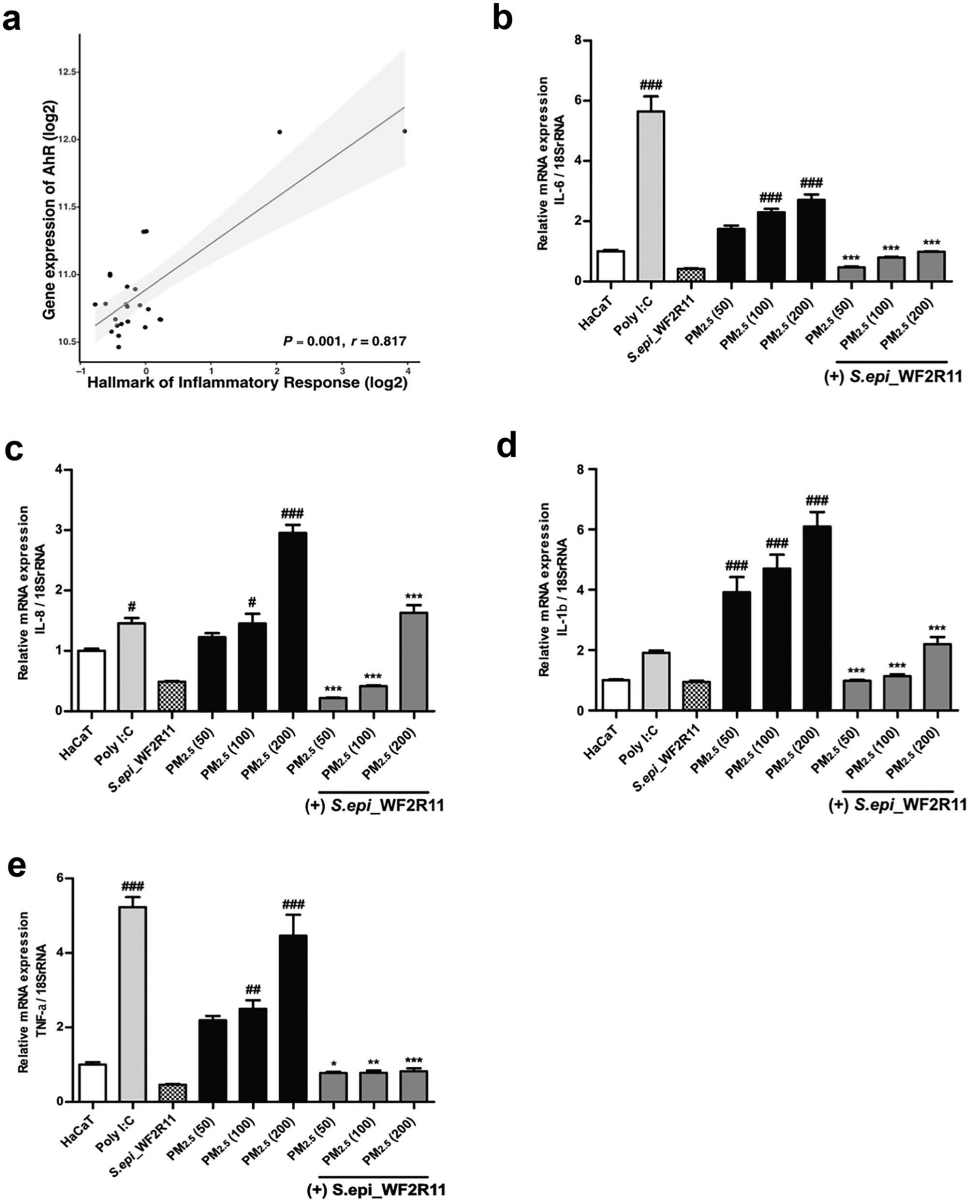
*Staphylococcus epidermidis* WF2R11 supernatant (Se solution) reduces the PM_2.5_-induced immune response. (**a**) Correlation analysis of *AhR* with genes associated with inflammatory responses reveals their positive correlation at the mRNA level. (**b**) Measurement of the mRNA level of *IL-6* cytokine relative to 18S rRNA. (**c**) Measurement of the mRNA level of *IL-8* cytokine relative to 18S rRNA. (**d**) Measurement of the mRNA level of *IL-1β* cytokine relative to 18S rRNA. (**e**) Measurement of the mRNA level of TNF-α cytokine relative to 18S rRNA. The mRNA level of each cytokine relative to 18S rRNA was measured after 12 h of PM_2.5_ treatment or PM_2.5_ and Se solution co-treatment for each concentration. Correlation was determined using Pearson’s correlation analysis. Bonferroni test for comparison between pairs was used to calculate statistical significance. ^#^*p* < 0.05, ^##^*p* < 0.01, ^###^*p* < 0.001, ns, non-significant; compared to normal HaCaT cells. *p < 0.05, **p < 0.01, ***p < 0.001, ns, non-significant; compared to each PM_2.5_-treated group. AhR, aryl hydrocarbon receptor

The AhR signaling pathway plays an important role in controlling the innate and adaptive immune response [44, 45]. The correlation between hallmark genes related to the immune response was analyzed according to the expression level of the *AhR* (*p* < 0.001, *r* = 0.817; Fig. 3a). AhR signaling is also closely related to the induction of oxidative stress, as shown in previous results. Similarly, an increase in oxidative stress led to an increase in the immune response (*p* < 0.001, *r* = 0.633; Online Resource 2a). To confirm these associations, the levels of immune cytokines were measured by treating HaCaT cells with PM_2.5_ at variable concentrations of 50, 100, and 200 µg/mL for 1, 2, and 4 h, respectively (Online Resource 1b–e). Subsequently, six skin-derived microbes that could significantly reduce the secretion of inflammatory cytokines induced via PM_2.5_ were screened (Online Resource 3). For screening, each concentration was fixed with a 10% concentration of the modified supernatant (Online Resource 1f). IL-1β, IL-6, IL-8, and TNF-α were selected as major screening factors that could contribute to the immune response of keratinocytes. Compared to the levels of four cytokine mRNAs in the untreated HaCaT cells, cytokine secretion was significantly reduced in cells treated with the supernatant derived specifically from the *S. epidermidis* WF2R11 culture medium (Online Resource 4a-d). The mRNA levels of inflammatory cytokines *IL-6*, *IL-8*, *IL-1β*, and *TNF-α*, were significantly reduced in the groups treated with Se solution compared to the respective PM_2.5_ concentrations (50, 100, 200 μg/mL)-treated groups (Fig. 3b–e). Also, upon comparing IL-1β, IL-6, IL-8, and interferon-gamma (IFN-γ) at the protein level, treatment with *S. epidermidis* WF2R11 supernatant significantly reduced protein abundance of all the cytokines mentioned above (Online Resource 4e-h). Taken together, *S. epidermidis* WF2R11 is a skin-bacterium that can significantly reduce the secretion of inflammatory cytokines induced by PM_2.5_.

### Se Solution Suppresses the Accumulation of ROS in HaCaT Cells

**Fig. 4:**
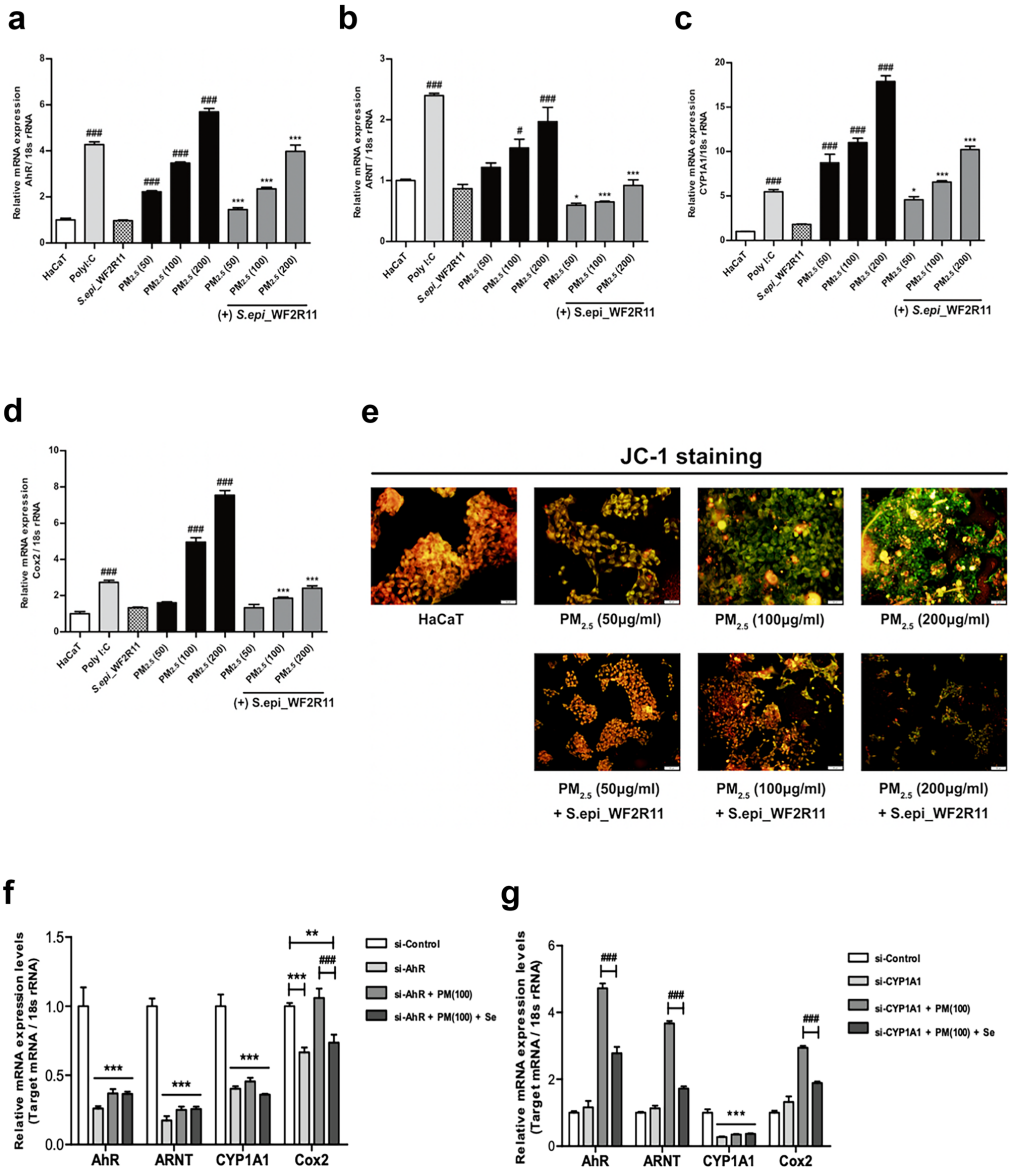
*Staphylococcus epidermidis* WF2R11 supernatant (Se solution) inhibits apoptosis by inhibiting the AhR signaling pathway and reducing ROS accumulation. Measurement of the mRNA level of (**a**) *AhR* relative to 18S rRNA, (**b**) *ARNT* relative to 18S rRNA, (**c**) *CYP1A1* relative to 18S rRNA, and (**d**) *Cox-2* relative to 18S rRNA. (**e**) Measurement of changes in JC-1 fluorescence expression when treated with Se solution compared to either normal HaCaT cells and 50, 100, or 200 μg/mL PM_2.5_ treatment groups. Scale bar, 50 μm. The mRNA level of *AhR*, *ARNT*, *CYP1A1*, and *Cox-2* relative to 18S rRNA was measured after 12 h of PM_2.5_ treatment or PM_2.5_ and Se solution co-treatment for each concentration. ^#^*p* < 0.05, ^###^*p* < 0.001; compared to normal HaCaT cells. *p < 0.05, ***p < 0.001; compared to each PM_2.5_-treated group. (**f**) Measurement of the *AhR*, *ARNT*, *CYP1A1*, *Cox-2* relative to 18S rRNA in Δ*AhR* HaCaT cells after 12 h of PM_2.5_ (100 μg/mL) treatment or PM_2.5_ (100 μg/mL) and Se solution co-treatment. (**g**) Measurement of the *AhR*, *ARNT*, *CYP1A1*, *Cox-2* relative to 18S rRNA in Δ*CYP1A1* HaCaT cells after 12 h of PM_2.5_ (100 μg/mL) treatment or PM_2.5_ (100 μg/mL) and Se solution co-treatment. Bonferroni test for comparison between pairs was used to calculate statistical significance. ^#^*p* < 0.05, ^##^*p* < 0.01, ^###^*p* < 0.001, ns, non-significant; comparison between PM_2.5_ treatment and PM_2.5_ and Se solution co-treatment. *p < 0.05, **p < 0.01, ***p < 0.001; compared to each si-control group

To determine whether the Se solution downregulates AhR signaling and reduces ROS production, HaCaT cells were treated with different concentrations of PM_2.5_ (50, 100, and 200 μg/mL) and inoculated with Se solution. The treatment with Se solution reduced the mRNA level of *AhR* by more than 30% at each PM_2.5_ concentration group (Fig. 4a). Regardless of the concentration of PM_2.5_ treatment, the level of *ARNT* mRNA was similar to that of the control group after treatment with the Se solution (Fig. 4b). As expected, the expression of *CYP1A1* was significantly reduced when inoculated with the Se solution than with only PM_2.5_ (Fig. 4c). In addition, activation of AhR may contribute to the upregulation of Cox-2 expression involved in ROS generation in the cytoplasm [44]. The expression of Cox-2 was significantly increased when PM_2.5_ was treated with 100 μg/ml or more. Moreover, when the Se solution was inoculated to the PM2.5-treated group of the same concentration, the expression level of Cox-2 was significantly reduced (Fig. 4d). Furthermore, JC-1 dye was used to measure the change in mitochondrial membrane potential due to ROS accumulation upon exposure to PM_2.5_. HaCaT cells stained with JC-1 showed a gradual loss of red J-aggregate fluorescence and cytoplasmic diffusion of green monomeric fluorescence after exposure to accumulated ROS in the cytoplasm. We confirmed that the cytoplasmic diffusion of green monomer fluorescence was significantly reduced when treated with the Se solution of each PM_2.5_ treatment group (Fig. 4e). Based on previous results, we hypothesized that *S.epidermidis* WF2R11 is caused by the inhibition of AhR signaling pathway due to the decrease in intracellular ROS accumulation via PM_2.5_. Therefore, to investigate the correlation between the *S. epidermidis* WF2R11 and the progression of the AhR signaling pathway, the *AhR* and the *CYP1A1* genes were silenced (Δ*AhR* and Δ*CYP1A1*) in HaCaT cells using siRNA.

First, siAhR treatment reduced the mRNA expression levels of *AhR*, *ARNT*, and *CYP1A1* genes to less than half in all other groups compared to the si-control group respectively. However, the *Cox-2* showed a lower mRNA expression level compared to other genes when treated with siAhR. In addition, the expression level of *Cox-2* in the group treated with siAhR and PM_2.5_ was almost similar to that of the si-control group. The mRNA expression was significantly reduced in the Se solution treatment group compared to the PM_2.5_ treatment group (Fig. 4f). Next, we determined whether *S. epidermidis* WF2R11 inhibits AhR alone or regulatea sub-mechanisms by silencing the *CYP1A1* gene. When *CYP1A1* was silenced, the mRNA expression levels of *AhR*, *ARNT*, and Cox-2 showed a similar trend to the previous results (Fig. 4a, b, d). However, the expression of *CYP1A1* gene was significantly lower than that of si-control mRNA in either the PM_2.5_ treatment group or the PM_2.5_ and Se solution treatment group (Fig. 4g). We also observed that the mRNA expression level of Cox-2 is independent of *CYP1A1* silencing and that the expression of Cox-2 gene is not directly regulated by *CYP1A1* gene expression. Therefore, the alleviation of PM_2.5_-derived ROS accumulation via the AhR signaling pathway of *S. epidermidis* WF2R11 would directly inhibit AhR and down-regulation the sub-gene *CYP1A1*. In addition, we assumed that the regulation of Cox-2 is correlated with other mechanisms according to AhR activity or is affected by the increased intracellular ROS due to increased *CYP1A1* expression.

### *Staphylococcus epidermidis* WF2R11 Inhibits Mitochondrial Apoptosis Induced by Accumulated Intracellular ROS

**Fig. 5:**
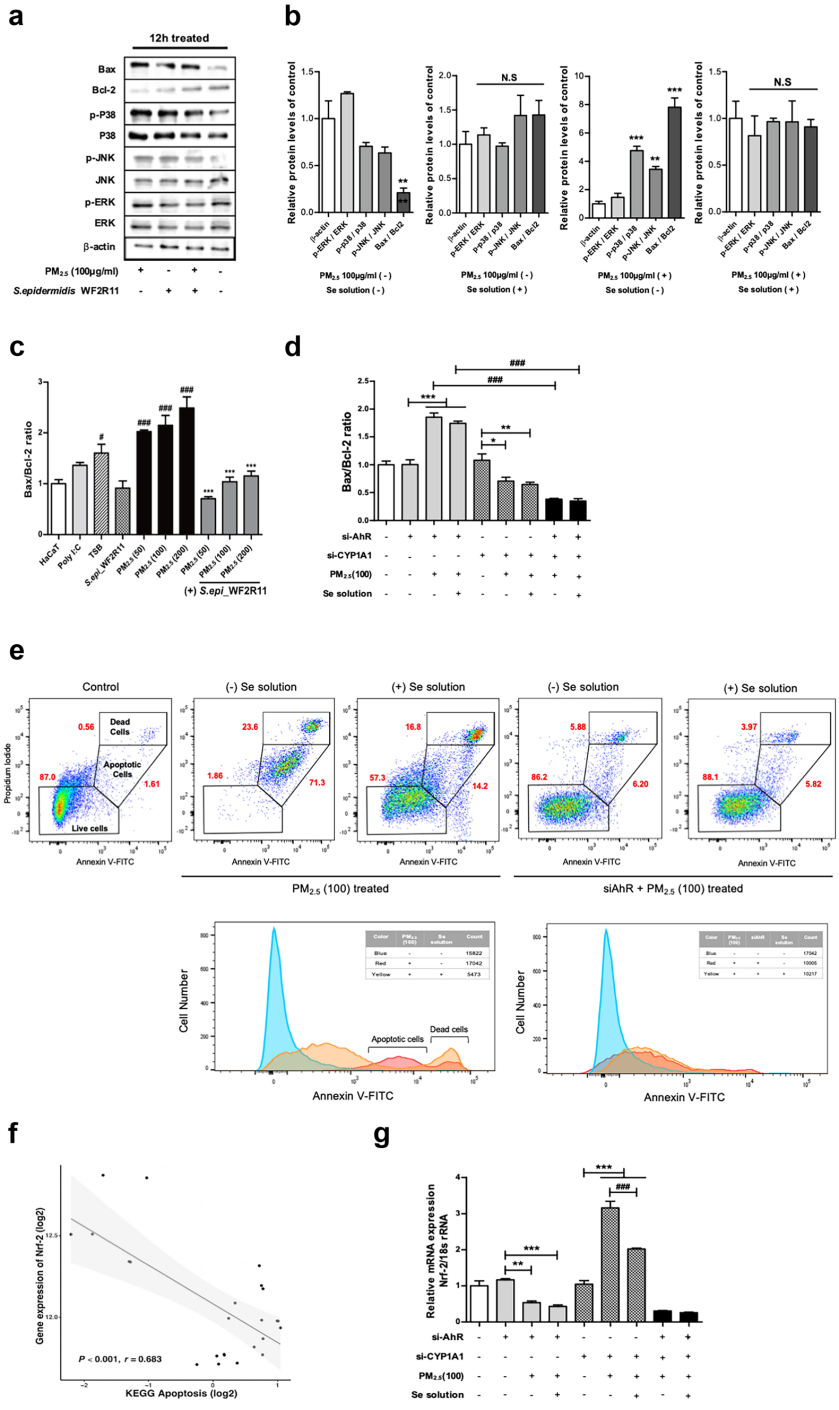
*Staphylococcus epidermidis* WF2R11 supernatant (Se solution) directly downregulates AhR and affects cell proliferation.(**a**) The protein expression of MAPK pathway-related proteins including p-p38, p38, p-JNK, JNK, p-ERK, ERK was detected via Western blot. (**b**) Quantitative analysis of p-ERK/ERK, p-p38/p38, and p-JNK/JNK based on Western blot results. **p < 0.01, ***p < 0.001, ns, non-significant; compared to internal control; β-actin. (**c**) Measurement of the relative *Bax*/Bcl-2 ratio level in response to mitochondrial-dependent cell death in the PM2.5 treatment group and Se solution treatment group. ^#^*p* < 0.05, ^###^*p* < 0.001; compared to normal HaCaT cells. ***p < 0.001; compared to each PM_2.5_-treated group. (**d**) Measurement of the relative *Bax/Bcl-2 ratio* in ΔAhR, Δ*CYP1A1,* and when both genes are silenced in HaCaT cells after 12 h of PM_2.5_ (100 μg/mL) treatment or PM_2.5_ (100 μg/mL) and Se solution co-treatment. *p < 0.05, **p < 0.01, ***p < 0.001; compared to each si-control group. ^###^*p* < 0.001; comparison between PM_2.5_ treatment and PM_2.5_ and Se solution co-treatment. (**e**) Visualization of the effect of Se solution treatment on apoptosis of siAhR-treated or untreated HaCaT cells during PM2.5 treatment using Annexin V-Pi staining. Representative scatterplot and histogram analysis data are shown. (**f**) Correlation analysis of Nrf-2 with KEGG-apoptosis reveals its negative correlation at the mRNA level. (**g**) Measurement of the mRNA level of *Nrf-2* relative to 18S rRNA in Δ*AhR*, Δ*CYP1A1*, and both genes are silenced in HaCaT cells. **p < 0.01, ***p < 0.001; compared to each si-control group. ^###^*p* < 0.001; comparison between PM_2.5_ treatment and PM_2.5_ and Se solution co-treatment. Bonferroni test for comparison between pairs was used to calculate statistical significance. Correlation was determined using Pearson’s correlation analysis

To investigate the mitochondrial apoptosis mechanism of PM_2.5_ mediated by AhR signaling, we assessed the phosphorylation levels of stress-activated protein kinases, such as c-Jun N-terminal kinase (JNK), extracellular signal-regulated kinase 1/2 (ERK), and p38 mitogen-activated protein kinase (p38 kinase). After treatment with either PM_2.5_ (100μg/ml) or PM_2.5_ (100 μg /ml) and Se solution for 12 h, the phosphorylation level of protein kinase was determined (Fig. 5a). Treatment with PM_2.5_ and Se solution simultaneously decreased both the expression of protein kinases and the degree of phosphorylation compared to PM_2.5_ only treatment. Furthermore, in the same conditions, the Bax protein expression decreased while the Bcl-2 protein expression increased in the group treated with the PM_2.5_ and Se solution compared to that treated with only PM_2.5_. We quantified the phosphorylation levels of each mitogen-activated protein kinase (MAPK), JNK, ERK, and p38 protein and calculated the Bax/Bcl-2 ratio for correlation with mitochondrial apoptosis mechanisms (Fig. 5b). Although the Bax/Bcl-2 ratio was significantly decreased in normal HaCaT cells compared to the control group (β-actin), the phosphorylation levels of p38 and JNK kinase and the Bax/Bcl-2 ratio were significantly increased following PM_2.5_ treatment.

In addition, Se solution had no effect on MAPKs phosphorylation or Bax/Bcl-2 ratio. Moreover, compared to other skin microbiota, *S.epidermidis* WF2R11 contributed to the observed decrease in mitochondrial *Bax* expression, which in turn decreased the Bax/Bcl-2 ratio caused by PM_2.5_ treatment (Online Resource 5a–c). However, in the group treated with PM_2.5_ and Se solution simultaneously, the change in the Bax/Bcl-2 ratio and the degree of phosphorylation of MAPKs were not significant compared to the control group. These results suggest that *S. epidermidis* WF2R11 decreased the Bax/Bcl-2 ratio in PM_2.5_-treated cells at each concentration, thereby significantly reducing mitochondrial-dependent apoptosis (Fig. 5c). Next, we determined whether PM_2.5_ enables mitochondrial apoptosis via AhR signaling pathway by partially blocking AhR signaling (Fig. 5d). The Bax/Bcl-2 ratio was almost similar in the siAhR-treated group (gray color) regardless of PM_2.5_ or PM_2.5_ and Se solution treatment. However, the siCYP1A1 treatment group (pattern) showed a significant decrease in the Bax/Bcl-2 ratio compared to the si-control group following PM_2.5_ treatment, and a more significant decrease compared to the PM_2.5_ alone treatment group when Se solution was added. Furthermore, when AhR signaling was blocked by treatment with both siAhR and siCYP1A1, the Bax/Bcl-2 ratio was significantly reduced regardless of PM_2.5_ and Se solution treatment. Our results suggest that *CYP1A1* is an important gene that mediates mitochondrial apoptosis signaling induced by PM_2.5_-induced intracellular ROS accumulation. In addition, *S. epidermidis* WF2R11 directly inhibits AhR to regulate the sub-mechanism of *CYP1A1-*induced sub-mechanisms. To further show that ROS accumulated in HaCaT cells via AhR signaling pathway induced mitochondrial apoptosis, we analyzed apoptosis by treating HaCaT cells with PM_2.5_ and PM_2.5_ and Se solution in siAhR-treated and untreated groups, respectively. We labeled cells with Annexin V-PI and observed the percentage of apoptotic cells using flow cytometry (Fig. 5e) and confirmed that PM_2.5_ alone treatment group significantly increased apoptosis compared to the control group. In addition, PM_2.5_ treatment group to which the Se solution was added showed significant decrease in apoptosis compared to the untreated group. Meanwhile, we treated siAhR-treated cells with PM_2.5_ and analyzed the ratio of apoptosis with and without Se solution. After siAhR treatment, no significant increase in apoptosis was observed in either group treated with PM_2.5_ regardless of Se solution treatment. In the PM_2.5_-treated group not treated with siAhR, treatment with Se solution shifted the histogram peak from apoptosis state to live state. However, in the PM_2.5_-treated group with siAhR, the histogram peaks of the two groups overlapped regardless of the Se solution. A significant positive correlation was also observed between the expression of *Cox-2* and the Fas Associated via death domain (*FADD*) gene (*p* < 0.01, *r* = 0.496; Online Resource 5d). In addition to *FADD*, the TNFRSF1A associated via death domain (*TRADD*) gene showed a significant positive correlation with *CYP1A1* (*p* < 0.01, *r* = 0.443; Online Resource 5e).

Ultimately, ROS accumulation via AhR signaling activation could increase *TRADD* gene expression due to mitochondrial-dependent apoptosis through TNF-α signaling (*p* < 0.05, *r* = 0.455; Online Resource 5f).

### *Staphylococcus epidermidis* WF2R11 Potentially Affects the Proliferation of HaCaT Cells by Activating Anti-oxidant Activity

**Fig. 6:**
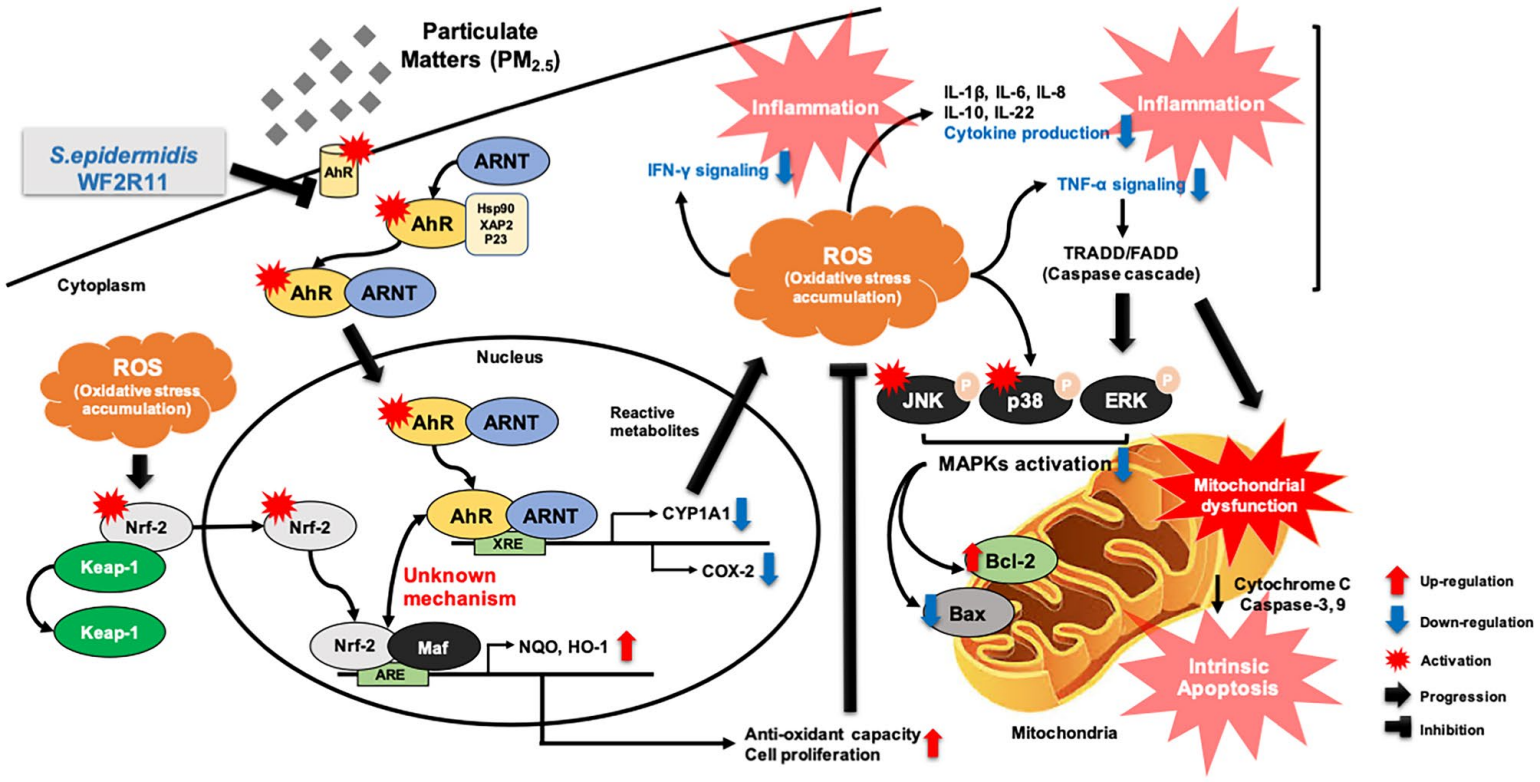
Schematic illustration showing the effect of *Staphylococcus epidermidis* WF2R11 on the intracellular changes induced by PM_2.5_. XRE, xenobiotic-binding factor; AhR, aryl hydrocarbon receptor; ARNT, AhR nuclear translocator; CYP1A1, cytochrome P450 family 1 subfamily A member 1; Cox-2, cyclooxygenase-2; Nrf-2, nuclear factor erythroid 2-related factor 2; ROS, reactive oxygen species; JNK, c-Jun N-terminal kinase; p38, p38 mitogen-activated protein kinase; ERK, extracellular signal-regulated kinase

PM_2.5_ is also known to induce DNA damage and apoptosis as well as arrest cell cycle G2/M transition due to mitochondrial dysfunction [46, 47]. The NF-E2-related factor 2 (Nrf2) and antioxidant response element (ARE) pathway are known to be involved in adaptation to oxidative stress through the upregulation of antioxidant activity and the expression of genes such as NAD(P)H-quinone dehydrogenase 1 (*NQO1*) and heme oxygenase-1 (*HO-1)* [48–50]. Therefore, transcriptomic analysis was used to investigate the effect of ROS production via AhR signaling activation on the Nrf-2–ARE signaling pathway. Using correlation analysis, the expression of *Nrf-2* was positively correlated with the hallmark genes of the AhR signaling pathway (*p* = 0.08, *r* = 0.360; Online Resource 6a). As a sub-mechanism of Nrf-2–ARE, the expression of *NQO1* (*p* = 0.07, *r* = 0.371; Online Resource 6b) and *HO-1* (*p* = 0.01, *r* = 0.485; Online Resource 6c) genes showed a significant positive correlation with AhR signaling. Consequently, increased expression of *Nrf-2* was ultimately associated with decreased apoptosis (*p* < 0.001, *r* = 0.683; Fig. 5f). The transcriptomic data showed changes in the expression of *Nrf2* and sub-genes (*NQO* and HO-1) with or without PM_2.5_ treatment by partially silencing AhR signaling pathway. The expression level of *Nrf2* was correlated with the activation of AhR signaling; meanwhile, when both *AhR* and *CYP1A1* were silenced, the expression level was significantly reduced (Online Resource 6d). Also, when the AhR expression was suppressed in *NQO1* and *HO-1*, the expression level of each gene was reduced to less than half regardless of *CYP1A1* expression (Online Resource 6e, f). Therefore, the direct inhibition of AhR was predicted to be a defense mechanism against ROS, which was promoted through AhR signaling and would eventually prevent apoptosis. As the suppression of apoptosis was expected to affect cell proliferation, Ki-67 immunohistochemistry was performed. The Ki-67 index was more than 70% in the groups inoculated with the five microbial supernatants after PM_2.5_ treatment; whereas, in the PM_2.5_ alone treatment group, the Ki-67 index was approximately 50% (Online Resource 6g). These results showed changes in *Nrf2* expression and confirmed the association of the Nrf-2 signaling pathway by upregulation of AhR signaling during PM_2.5_ treatment. Moreover, we confirmed the involvement of *S. epidermidis* WF2R11 in HaCaT cell proliferation by participating in AhR-Nrf2 signaling. Thus, we next silenced *AhR* and *CYP1A1* and observed *Nrf2* gene expression following treatment with PM_2.5_ (100 μg/ml), Se solution, and PM_2.5_ (100 μg/ml) and Se solution. The expression of *Nrf2* was significantly decreased in the *AhR* silenced group (gray color) regardless of Se solution treatment with PM_2.5_ compared to the control. However, in the group (pattern) in which *CYP1A1* was silenced, the expression of *Nrf2* was increased more than threefold compared to the control when PM_2.5_ was treated. Meanwhile, when comparing the PM_2.5_-treated and the Se solution-treated groups, the expression of *Nrf2* was significantly reduced in the Se solution-treated group, although the group in which both *AhR* and *CYP1A1* were silenced (black color) showed almost no expression of *Nrf2* regardless of PM_2.5_ and Se solution treatment (Fig. 5g). Taken together, these results suggest that *S. epidermidis* WF2R11 effectively inhibited ROS production through AhR signaling and caused a decrease in apoptosis. In this process, the Nrf-2 signaling pathway, a part of AhR signaling, was directly/indirectly upregulated based on AhR signaling and ROS generation, affecting the regulation of cell proliferation as well as mitochondrial-dependent cell death (Fig. 6).

## Discussion

This study demonstrated the effect of PM_2.5_ exposure on the AhR pathway and mitochondrial dysfunction-dependent apoptosis. To the best of our knowledge, this is the first study to analyze how a member of skin microbiota can downregulate the AhR pathway, which leads to the inhibition of skin inflammation and ROS production. We specifically investigated the effect of *S. epidermidis* WF2R11 on the progression of the AhR pathway. The main mechanism by which PM_2.5_ induces cellular oxidative stress is through the generation of ROS in skin keratinocytes [51]. PM_2.5_ directly increases the level of ROS production and free radicals on the surface of skin keratinocytes, indicating potent redox activity in PAHs [52, 53]. In addition, oxy-PAH reportedly promotes the oxidation of nucleic acids, proteins, and lipids more than PAH, which can cause severe redox stress in cells and tissues [54, 55]. Water-soluble PAHs such as BaP, unlike other organic compounds, tend to oxidize rapidly [56], and oxy-PAHs show increased cell permeability and AhR reactivity [26]. Initiation of AhR-mediated signaling by PAHs or oxy-PAHs is first transferred to the nucleus upon binding of the PM_2.5_-binding AhR complexes to ARNT [26, 57]. It was evident that, when PM_2.5_ acted as a ligand for AhR, both the AhR and ARNT mRNA levels increased. Our results demonstrated that although *S. epidermidis* WF2R11 did not directly affect AhR, it reduced *AhR* and *ARNT* mRNA levels. It was initially speculated that the reduced expression of *AhR* and *ARNT* is caused by the metabolites secreted by *S. epidermidis* WF2R11, thereby affecting the formation of AhR/ARNT complexes. However, when *AhR* was knocked down in HaCaT cells, *ARNT* and *CYP1A1* expression was inhibited regardless of treatment with PM_2.5_ or the *S. epidermidis* WF2R11 supernatant. Although previous hypotheses have speculated that the *S. epidermidis* WF2R11 metabolites inhibit the formation of the AhR/ARNT complex, this finding demonstrated that AhR activation to PAH is a prerequisite for *ARNT* and *CYP1A1* expression. We therefore suggest that the *S. epidermidis* WF2R11 metabolites may operate by suppressing AhR activity on PAHs or act as a competitive ligand inhibitor, rather than directly inhibiting the formation of the AhR/ARNT complexes. AhR/ARNT heterodimers bind to xenobiotic responsive elements and activate the transcription of sub-target genes such as *CYP1* [58]. CYP1 is a key factor capable of promoting ROS accumulation in the cytoplasm, concomitantly causing oxidative stress [59, 60]. In addition, CYP enzymes induce ROS accumulation in the cytoplasm by damaging keratinocytes and altering DNA formation [25, 37, 61]. The mRNA level of *CYP1A1* was significantly increased after PM_2.5_ treatment, while *S. epidermidis* WF2R11 was involved in the inhibition of AhR activity, thereby reducing the expression of *CYP1A1*. Thus, the AhR/ARNT complex affects the activity of the CYP1 enzyme, thereby increasing the expression of *CYP1A1* [39, 62]. In addition, cytoplasmic ROS production by *Cox-2* overexpression in HaCaT cells critically exerts oxidative stress on various organelles, including mitochondria [59, 63]. Signaling due to AhR activity was associated with an increase in *CYP1A1* and in *Cox-2* expression [64]. In particular, when AhR was silenced, the expression of *Cox-2* was significantly reduced, which was restored following PM_2.5_ treatment to the same level as that of si-control. However, despite silencing *CYP1A1*, signal transduction of AhR caused an increase in the expression of *Cox-2*, which suggests that the expression of *Cox-2* is determined somewhat independently of the sub-mechanism of *CYP1A1*, and its expression may be regulated by AhR activity. Moreover, PAH and oxy-PAH result in differential ROS generation and Ca^2+^ perturbation and promote electrophysiological instability, which increases mitochondrial inner membrane permeability. Furthermore, an increase in ROS not only produce a cytotoxic effect, but also activate MAPK pathways, including JNK, ERK, and p38 MAPK, which ultimately lead to mitochondrial stress and excessive free radical accumulation, resulting in mitochondrial dysfunction and apoptosis (i.e., the intrinsic pathway of apoptosis) [65–69]. We visualized mitochondrial superoxide and membrane potential after PM_2.5_ and Se solution treatment via fluorescent staining (Mito-Sox, JC-1), suggesting that the metabolite of *S. epidermidis* WF2R11 is effective in suppressing PM_2.5_-induced oxidative stress. Furthermore, we demonstrated that the metabolites of S. epidermidis WF2R11 can significantly reduce phosphorylation of p38 kinase and JNK among ROS-activated MAPK subgroups. Bax is phosphorylated by stress-activated p38 kinase and/or JNK and phosphorylation of Bax leads to mitochondrial translocation prior to apoptosis. The mitochondrial translocation of pro-apoptotic Bax can act as apopotosis stimulators or conditions that induce mitochondrial apoptosis [70, 71]. PM_2.5_-induced apoptosis upregulates *Bax* and downregulates *Bcl-2* [68, 72–74]; therefore, a decrease in the *Bax/Bcl-2* ratio suggests a decrease in mitochondrial dysfunction-dependent apoptosis in the *S. epidermidis* WF2R11-treated group. Although the mechanism by which increased ROS can activate ERK, JNK and p38 MAPK remain unclear [75], we found that groups treated with *S. epidermidis* WF2R11 did not show any significant difference in the regulation of *Bcl-2* but exhibited downregulated *Bax* expression. In addition, since the PM_2.5_-derived ROS-induced mitochondrial apoptosis mechanism is increased through the AhR signaling pathway, the change in the Bax/Bcl-2 ratio was confirmed after suppressing the *AhR* or *CYPA1* gene, or both. We confirmed that the key gene for PM2.5-derived ROS increase was the expression of *CYP1A1*, while *S. epidermidis* WF2R11 suppressed AhR, the upper gene of *CYP1A1*, to reduce PM_2.5_-derived ROS. Transcriptome analysis showed that the AhR signaling pathway not only increased the expression of *CYP1A1* but also contributed to the activation of TNF-α signaling to induce mitochondrial apoptosis, or upregulate *Cox-2*, thereby contributing to the activity of p38 and JNK MAPKs to enable translocation of mitochondrial *Bax*. Taken together, excessive oxidative stress due to the AhR pathway specifically affects the expression level of *Bax* via phosphorylation of MAPKs and may be a major contributor to mitochondrial dysfunction [76–78]. The reduction of oxidative stress, resulting from inhibition of AhR pathway progression, may have an inhibitory effect on the upregulation of *Bax*, which suggests AhR as an important receptor that leads to apoptosis by causing mitochondrial dysfunction. Furthermore, the metabolites of *S. epidermidis* WF2R11 can inhibit the progression of ROS-mediated damage by reducing AhR activity*.* Endogenous ROS can also cause structural damage and cell degradation, leading to apoptosis in HaCaT cells [33]. Previous studies showed that mitochondrial dysfunction caused by PM_2.5_ triggers an inflammatory cascade [34]. Activated Toll-like receptors initiate the NF-κB pathway and secrete inflammatory cytokines such as IL-1α, IL-1β, IL-6, IL-8, and TNF-α [79]. The secretion of these cytokines promotes the rapid activity of inflammatory reactions. Activated NF-κB initiates an inflammatory cascade, which causes the skin barrier to collapse, aggravating skin inflammation and leading to apoptosis [17, 80, 81]. The finding of this study suggests that AhR regulation of microbial metabolites is expected to help alleviate these inflammatory responses. Finally, we confirmed whether *S. epidermidis* WF2R11 effectively reduced apoptosis and inflammatory responses from PM_2.5_-induced oxidative stress via AhR-mediated Nrf2 signaling. Previous studies have suggested two different mechanisms for the activation of *Nrf2* by AhR: direct transcriptional activation of *Nrf2* following AhR signaling activation or ROS generation by *CYP1A1* induction [82, 83]. Among these two hypotheses, we investigated the pathway by which metabolites of *S. epidermidis* WF2R11 influence initiation of Nrf2 signaling. AhR silencing significantly reduced the expression level of *Nrf2* and sub-genes such as *NQO1* and *HO-1* regardless of PM_2.5_ or *S. epidermidis* WF2R11 treatment. However, when *CYP1A1* was silenced, while treating with PM_2.5_, the expression of *Nrf2* gene was significantly increased, whereas when PM_2.5_ and *S. epidermidis* WF2R11 were co-treated, the expression of *Nrf2* was significantly decreased.

These results indicate that AhR, not *CYP1A1*, has direct transcriptional activation function for Nrf2 signal and that two other pathways of AhR may be activated simultaneously, one to increase cellular ROS and the other to trigger an antioxidant response. Furthermore, the decrease in expression *Nrf2* following inhibition of AhR may possibly reduce the activation signal for other ROS generation mechanisms involved in the AhR signaling pathway. Conversely, we consider that the reason for the increase in *Nrf2* during PM_2.5_ treatment despite the silencing of *CYP1A1* is probably attributed to ROS generation associated with the increased expression of *Cox-2* based on the activity of AhR. Thus, the direction of pathway progression of AhR will depend on either the ligand, intracellular ROS accumulation, or additional factors, which will determine the agonist or compensatory antioxidant AhR pathway.

Taken together, these findings provide new insights into the potential application of skin microbiome interventions in clinical practice. However, this study could not comprehensively explore the mechanism by which the metabolites of *S. epidermidis* WF2R11 can inhibit the activation of AhR. Therefore, further research is required to study the effects of metabolites secreted by the skin microbiome on the AhR signaling pathway. We will also investigate the association of AhR-Trp metabolism and explore the functional significance of microbial Trp metabolites in skin inflammation. Future studies should harbor the prospect of overcoming these limitations and treating skin diseases caused by PM_2.5_ exposure.

## Supplementary Information

Below is the link to the electronic supplementary material.

Supplementary file1 (DOCX 3184 KB)

## Data Availability

All processed gene expression data used in this study were procured from the Gene Expression Omnibus under accession number GSE107871.
