# Supplementary material for: Staphylococcus epidermidis WF2R11 Suppresses PM2.5-Mediated Activation of the Aryl Hydrocarbon Receptor in HaCaT Keratinocytes
Source: Probiotics Antimicrob Proteins. 2022 Jun 21;14(5):915–33. doi: 10.1007/s12602-022-09922-8 (PMC9474527; doi:10.1007/s12602-022-09922-8)
Supplement: Supplementary file 1 — Supplementary file1 (DOCX 3184 KB) [file 12602_2022_9922_MOESM1_ESM.docx]

# *Staphylococcus epidermidis* WF2R11 suppresses PM_2.5_-mediated activation of the aryl hydrocarbon receptor in HaCaT keratinocytes

# *Probiotics and Antimicrobial Proteins*

Eulgi Lee^1^, Hyeok Ahn^1^, Shinyoung Park^2^, Gihyeon Kim^1^, Hyun Kim^1^, Myung-Giun Noh^1^, Yunjae Kim^1^, Jae-sung Yeon^2^, Hansoo Park^1,2*^

^1^Department of Biomedical Science and Engineering, Gwangju Institute of Science and Technology (GIST), Gwangju 61005, Republic of Korea

^2^Genome and Company, Pangyo-ro 253, Bundang-gu, Seoungnam-si, Gyeonggi-do 13486, Republic of Korea

***Correspondence**: Hansoo Park, Department of Biomedical Science and Engineering, Gwangju Institute of Science and Technology (GIST), Gwangju 61005, Republic of Korea. E-mail: [hspark27@gist.ac.kr](mailto:hspark27@gist.ac.kr)

**Online Resource 1. PM_2.5_ dose setting based on AQI index in this study**

| AQI category | Index value | Previous Breakpoints (1999 AQI, μg/m^3^, 24-h average) | Revised Breakpoints (μg/m^3^, 24-h average) | Experimental Dosage |
| --- | --- | --- | --- | --- |
| Good | 0–50 | 1. 15.0 | 0.0–12.0 | - |
| Moderate | 51–100 | > 15.0 -40 | 12.1–35.4 | PM_2.5_ **(50 μg/mL, moderate)** |
| Unhealthy for sensitive groups | 101–150 | > 40 – 65 | 35.5–55.4 |  |
| Unhealthy | 151–200 | > 65–150 | 55.5–150.4 | PM_2.5_ **(100 μg/mL, unhealthy)** |
| Very unhealthy | 201–300 | > 150–250 | 150.5–250.4 | PM_2.5_ **(200 μg/mL, hazardous)** |
| Hazardous | 301–400 | > 250–350 | 250.5–350.4 |  |
|  | 401–500 | > 350–500 | 350.5–500 |  |

**
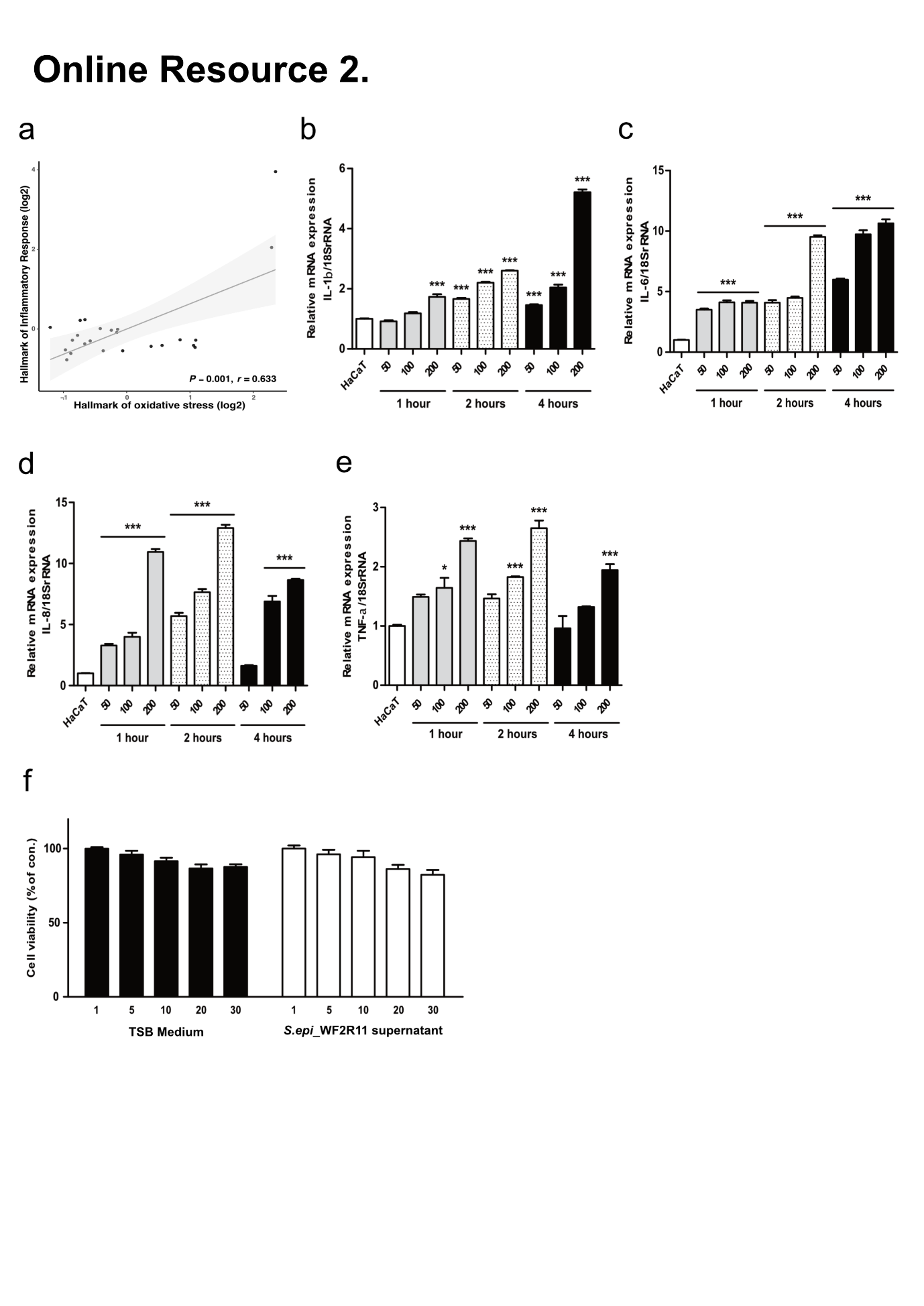
**

**Online Resource 2. Analysis of changes in the immune response of HaCaT cells to PM_2.5_ treatment and evaluation of the stability of Se solution.** (**a**) Correlation analysis of the hallmark genes of the inflammation response with the hallmark genes of oxidative stress reveals a positive correlation. Measurement of the mRNA levels of (**b**) *IL-1β*, (**c**) *IL-6*, (**d**) *IL-8*, or (**e**) TNF-α cytokines relative to 18S rRNA upon treatment with PM_2.5_ at respective concentrations of 50, 100, and 200 μg/mL for 1, 2, and 4 h, respectively. (**f**) Cell viability measurement using the supernatant of *Staphylococcus epidermidis* WF2R11 (i.e., Se solution) compared to TSB medium. Correlation was determined using Pearson’s correlation analysis. Bonferroni test for comparison between pairs was used to calculate statistical significance. **p* < 0.05, ***p* < 0.01, ****p* < 0.001, ns, non-significant; compared to each normal HaCaT cells.

**Online Resource 3. List of the skin bacteria used in this study**

| Strain | Closest match (16S rRNA gene similarity) | Hemolysis |
| --- | --- | --- |
| WF2R11 | *Staphylococcus epidermidis* (99%) | γ |
| WF1R12 | *Dermacoccus nishinomiyaensis* (99%) | γ |
| WF1T10 | *Staphylococcus epidermidis* (99%) | γ |
| WF3L4 | *Kocuria palustris* (100%) | γ |
| WF7R9 | *Staphylococcus hominis* (99%) | γ |
| WF9R3 | *Paracoccus yeei* (99%) | γ |

**
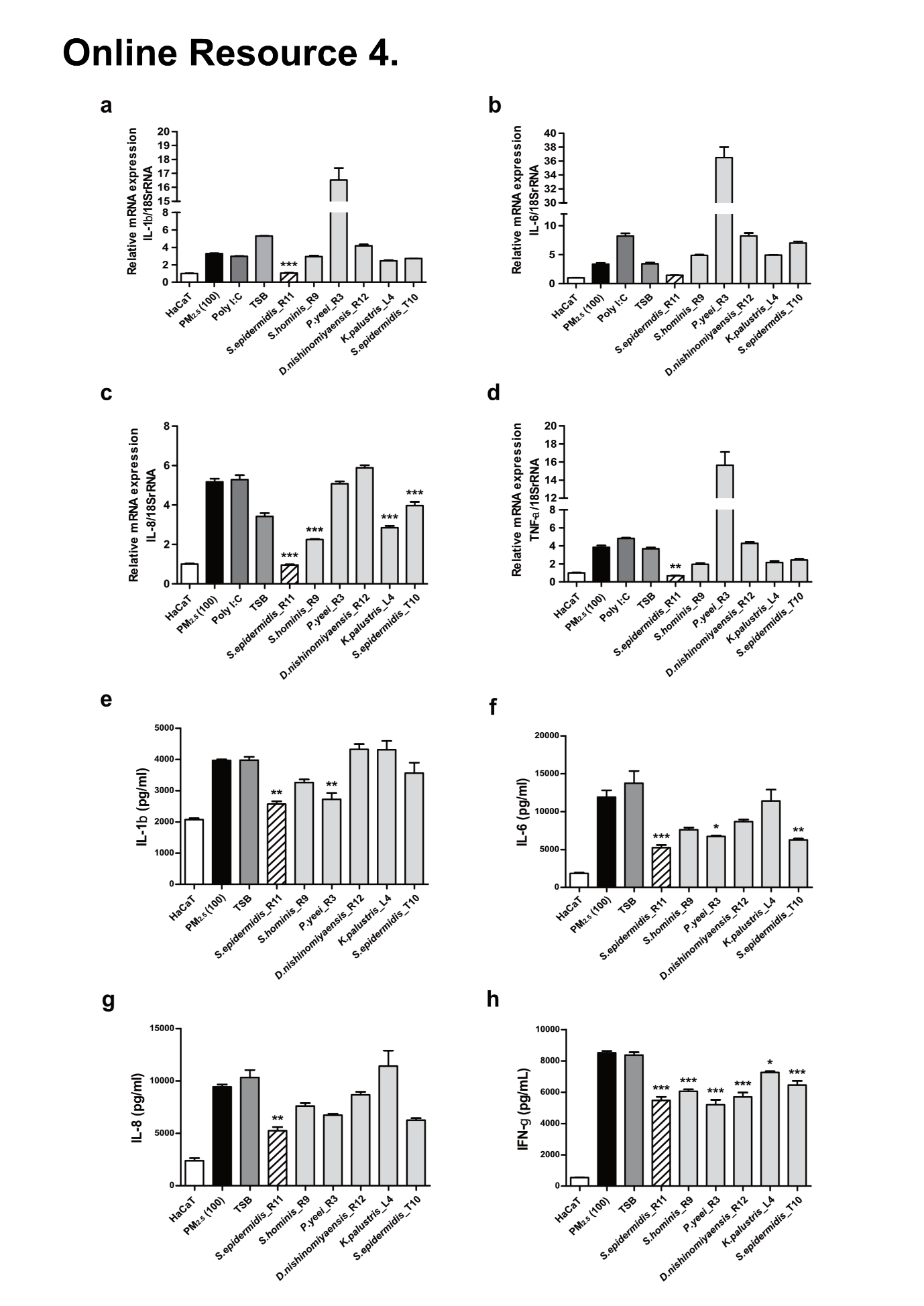
**

**Online Resource 4. Immune response measurement via qPCR and ELISA for screening *Staphylococcus epidermidis* WF2R11 supernatant.** Measurement of the mRNA level of (**a**) *IL-1β*, (**b**) *IL-6*, (**c**) *IL-8*, or (**d**) TNF-α cytokines relative to 18S rRNA after treatment of six skin-derived microbial supernatants in the PM_2.5_ treatment group. Measurement of the concentration of (**e**) *IL-1β*, (**f**) *IL-6* (**g**) *IL-8*, or (**h**) *IFN-γ* cytokine using ELISA after treatment with six skin-derived microbial supernatants in the PM_2.5_ treatment group. Bonferroni test for comparison between pairs was used to calculate statistical significance. **p* < 0.05, ***p* < 0.01, ****p* < 0.001, ns, non-significant; compared to each PM_2.5_ treatment group.


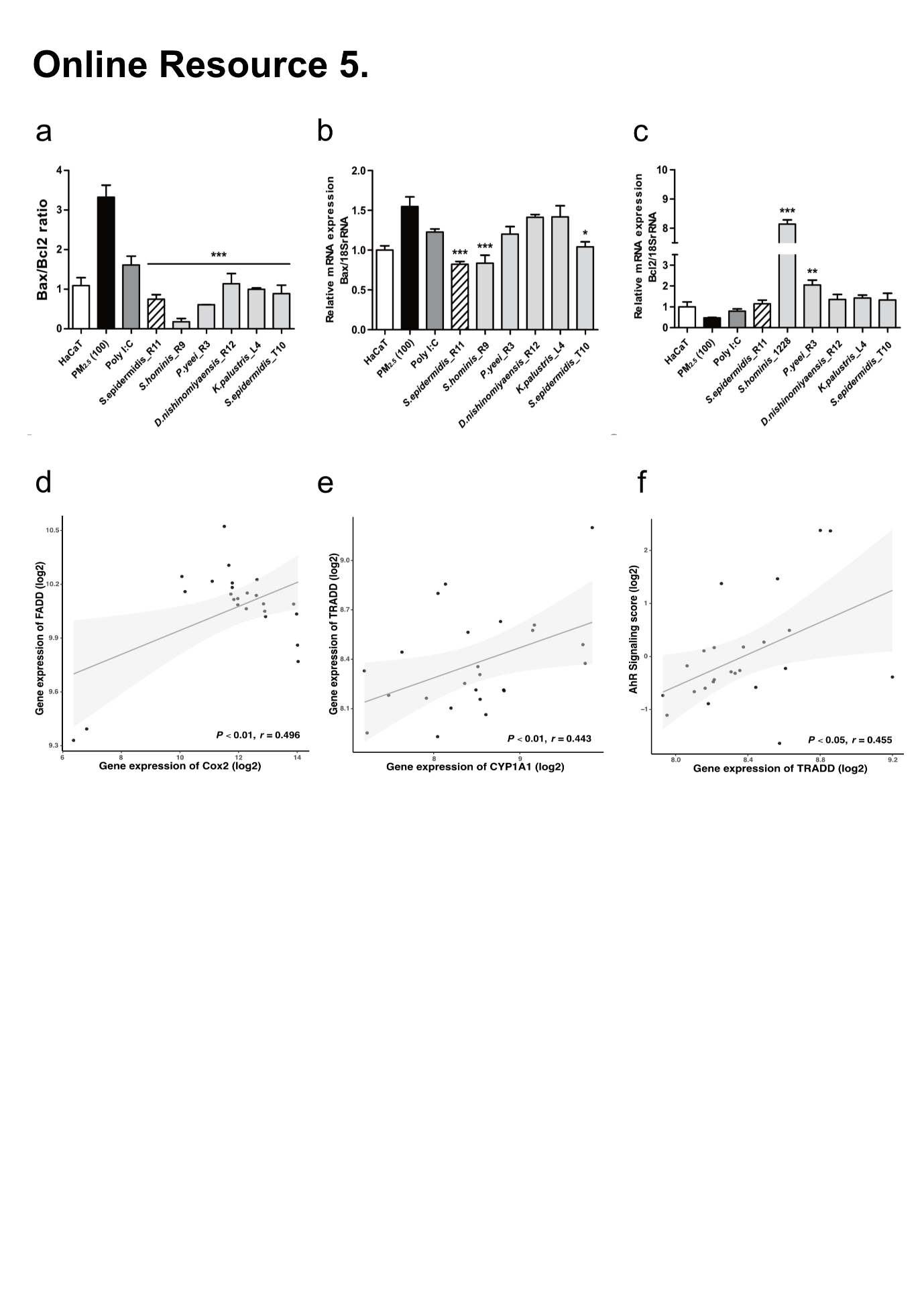


**Online Resource 5. Analysis of the association between AhR signaling and TNF-α signaling-dependent apoptosis.** (**a**) Analysis of the change in the relative *Bax/Bcl-2* ratio to mitochondrial-dependent cell death upon treatment with supernatants from six skin microbes. Measurement of the mRNA level of (**b**) *Bax* and (**c**) *Bcl-2* relative to the 18S rRNA after treatment with six skin-derived microbial supernatants in the PM_2.5_ treatment group. Correlation analysis of (**d**) FADD with *Cox-2*, (**e**) *TRADD* with *CYP1A1*, and (**f**) hallmark genes of AhR signaling with *TRADD* expression reveal their respective positive correlations at the mRNA level. Correlation was determined using Pearson’s correlation analysis. **p* < 0.05, ***p* < 0.01, ****p* < 0.001, ns, non-significant; compared to each PM_2.5_ treatment group.


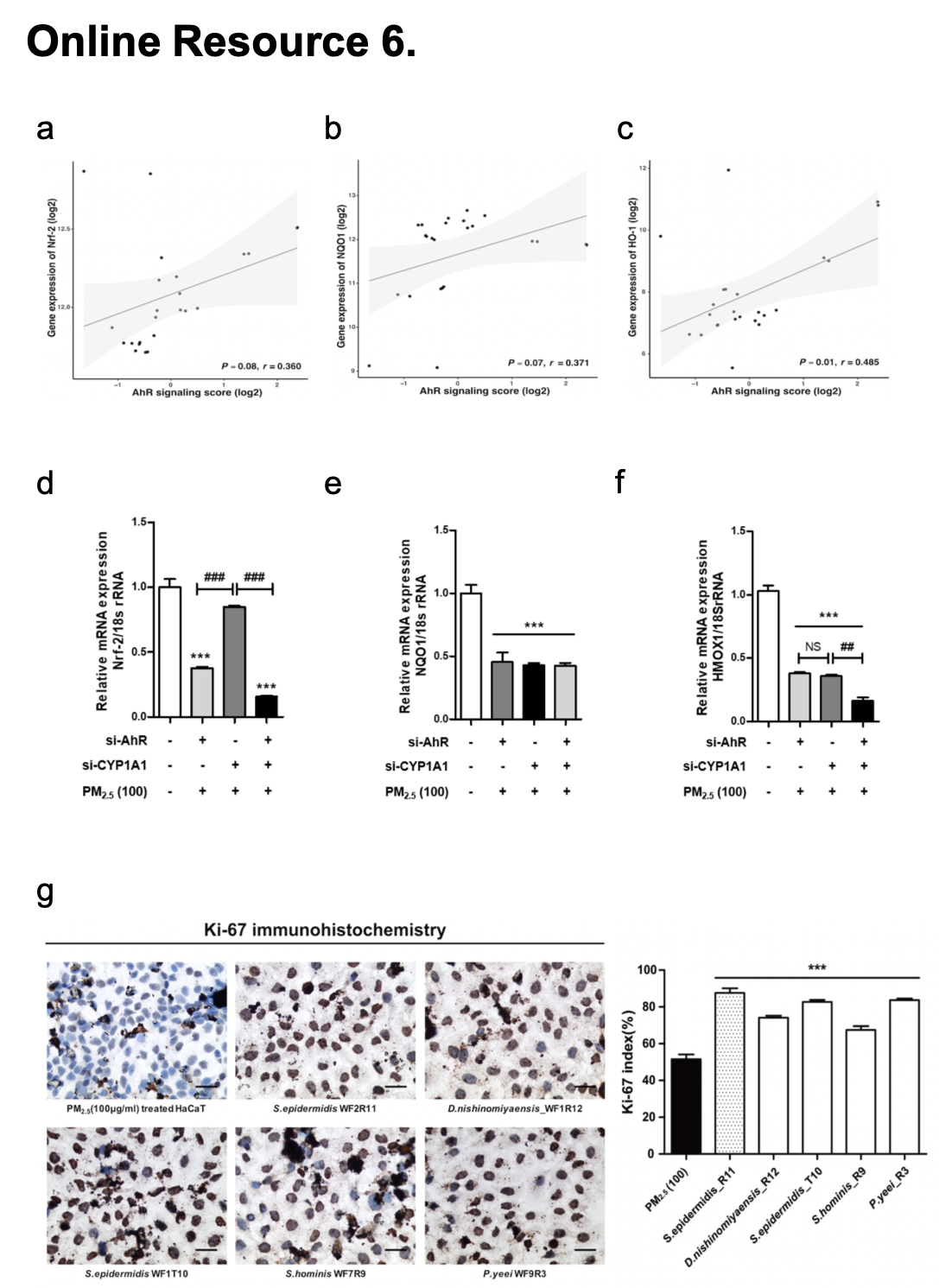


**Online Resource 6. Analysis of the correlation of antioxidant gene expression to AhR signaling and its effect on apoptosis.** Correlation analysis of (**a**) *Nrf-2*, (**b**) *NQO-1*, and (**c**) *HO-1* with the hallmark genes of AhR signaling reveal their respective positive correlations at the mRNA level. Correlation was determined using Pearson’s correlation analysis. (**d**) mRNA levels of Nrf-2, NQO-1 and HO-1 relative to 18S rRNA in Δ*AhR* and Δ*CYP1A1* HaCaT cells after 12 hours of PM_2.5_ (100 μg/mL) treatment. **p* < 0.05, ***p* < 0.01, ****p* < 0.001; compared to each si-control group. ^##^*p* < 0.01, ^###^*p* < 0.001, ns, non-significant; comparison between siAhR-treated and siAhR-untreated groups. (**g**) Analysis of Ki-67 immunohistochemistry pattern and measurement of Ki-67 index at 40× magnification upon treatment with five microbial supernatants compared to the PM_2.5_ treatment group. Scale bar, 50 μm. Bonferroni test for comparison between pairs was used to calculate the statistical significance. The Ki-67 index sets the PM_2.5_ treatment group as a control. ****p* < 0.001; compared to PM_2.5_ (100μg/mL) treatment HaCaT group. Bonferroni test for comparison between pairs was used to calculate statistical significance.

**Online Resource 7. List of primer pairs used in this study**

| Primer name | Sequence | Pairs |
| --- | --- | --- |
| 18s rRNA | 5ʹ-AGAAACGGCTACCACATCCA-3ʹ | Forward |
|  | 5ʹ-CCCTCCAATGGATCCTCGTT-3ʹ | Reverse |
| IL-1β | 5ʹ-TGAGCTCGCCAGTGAAATGA-3ʹ | Forward |
|  | 5ʹ-AGATTCGTAGCTGGATGCCG-3ʹ | Reverse |
| IL-6 | 5ʹ-AGTGAGGAACAAGCCAGAGC-3ʹ | Forward |
|  | 5ʹ-AGCTGCGCAGAATGAGATGA-3ʹ | Reverse |
| IL-8 | 5ʹ-GGTGCAGTTTTGCCAAGGAG-3ʹ | Forward |
|  | 5ʹ-TTCCTTGGGGTCCAGACAGA-3ʹ | Reverse |
| Bcl-2 | 5ʹ-TCATGTGTGTGGAGAGCGTC-3ʹ | Forward |
|  | 5ʹ-GCCGTACAGTTCCACAAAGG-3ʹ | Reverse |
| Bax | 5ʹ-CTTCAGGGGATGATTGCCGC-3ʹ | Forward |
|  | 5ʹ-TGTCCAGCCCATGATGGTTC-3ʹ | Reverse |
| TNF-α | 5ʹ-CTCTTCTGCCTGCTGCACTTTG-3ʹ | Forward |
|  | 5ʹ-ATGGGCTACAGGCTTGTCACTC-3ʹ | Reverse |
| AhR | 5ʹ-TGTATCAGTGCCAGCCAGAA-3ʹ | Forward |
|  | 5ʹ-TGACGGATGATGAAGTGGCT-3ʹ | Reverse |
| ARNT | 5ʹ-AGGTCGGATGATGAGCAGAG-3ʹ | Forward |
|  | 5ʹ-GCACTACAGGTGGGTACCAT-3ʹ | Reverse |
| CYP1A1 | 5ʹ-GCTAGGCTCCTGGGATCGAG-3ʹ | Forward |
|  | 5ʹ-GTTCAGGGCAAGGTTCCAGTCA-3ʹ | Reverse |
| Cox-2 | 5ʹ-AGGAGGTCTTTG GTCTGGTG-3ʹ | Forward |
|  | 5ʹ-TAGCCTGCTTGTCTGGAACA-3ʹ | Reverse |
| Nrf2 | 5′-ATGGCCTCCCTGTACCACATC-3′ | Forward |
|  | 5′-TGTTGCGCTCAATCTCCTCCT-3′ | Reverse |
| NQO-1 | 5′-CGCAGACCTTGTGATATTCCAG-3′ | Forward |
|  | 5′-CGTTTCTTCCATCCTTCCAGG-3′ | Reverse |
| HO-1 | 5′-ATGGCCTCCCTGTACCACATC-3′ | Forward |
|  | 5′-TGTTGCGCTCAATCTCCTCCT-3′ | Reverse |
